# Mannosylated nanocarriers: a precision targeting strategy for tumors and infectious diseases

**DOI:** 10.3389/fphar.2026.1744292

**Published:** 2026-03-25

**Authors:** Bo Gao, Zhiwen Wang, Wei Tan

**Affiliations:** 1 Weifang People’s Hospital, Shandong Second Medical University, Weifang, China; 2 School of Clinical Medicine, Shandong Second Medical University, Weifang, China

**Keywords:** antibiotic delivery, cascade-targeting drug delivery systems, drug delivery system, mannose receptor, nanoparticles, targeted cancer therapy

## Abstract

Conventional chemotherapy often suffers from systemic toxicity, suboptimal efficacy, and drug resistance due to the non-specific distribution of drugs in the treatment of tumors and infectious diseases. Mannose-functionalized nanodelivery systems present a promising precision-targeting strategy to overcome these challenges. The core of this strategy lies in leveraging the high expression of C-type mannose receptors (MR, CD206) on the surface of various tumor cells, tumor-associated macrophages (TAMs), and antigen-presenting cells (APCs). Not only do mannose-functionalized nanocarriers achieve precise drug delivery to specific cells via receptor-mediated endocytosis, but they also actively modulate the tumor immune microenvironment. This modulation occurs by activating antigen presentation mechanisms, thereby enhancing the host’s immune response. Consequently, this system exhibits immense potential for intervention in both malignant tumors and infectious diseases. This review systematically summarizes nanodelivery platforms integrated with mannose-targeted strategies. Furthermore, it explores their recent advances and future application perspectives in combination with diverse therapeutic modalities, including chemotherapy, photodynamic therapy, and immunotherapy.

## 1 Introduction

Malignant tumors and infectious diseases represent major threats to human health. Despite the widespread clinical application of conventional treatments such as surgery, chemotherapy, radiotherapy, and antibiotics, significant limitations persist. These include poor drug selectivity, severe systemic toxicity, low bioavailability, and the escalating challenge of drug resistance. Consequently, the development of novel therapeutic approaches capable of precisely delivering therapeutic agents to pathological sites while minimizing off-target effects has become an urgent imperative in modern biomedical research.

Nanoparticles (NPs) and other nanostructures, owing to their ultra-small size (1–100 nm) (Chen et al., 2022), high surface-to-volume ratio, and favorable cell permeability, serve as excellent drug delivery vehicles for disease treatment. Nanomedicine, particularly nanocarrier-based drug delivery systems (DDSs), offers revolutionary pathways to address these challenges. By leveraging unique size-dependent effects [such as the enhanced permeability and retention (EPR) effect], high drug-loading capacity, and readily modifiable surface properties, nanocarriers can significantly improve the pharmacokinetic behavior of drugs and enable controlled drug release (Lv et al., 2016). Metal-based or carbon-based nanomaterials, due to their high electron mobility and favorable photoelectrochemical activity, are frequently employed in biosensors (Jia et al., 2020) for disease or biomarker detection. Furthermore, nanomaterials with magnetic or optical properties are widely utilized in cancer diagnosis and therapies such as magnetic hyperthermia or photothermal therapy (Zhao et al., 2020; Zhou et al., 2022). However, passive targeting strategies relying solely on the EPR effect exhibit significant heterogeneity across different tumor types and patients, limiting their universal clinical efficacy. Additionally, unresolved issues such as potential cytotoxicity, low bioavailability, and *in vivo* metabolic degradation necessitate further investigation. Therefore, surface modification of nanocarriers with specific ligands for active targeting of diseased cells has emerged as a critical strategy to enhance therapeutic precision.

**FIGURE 1:**
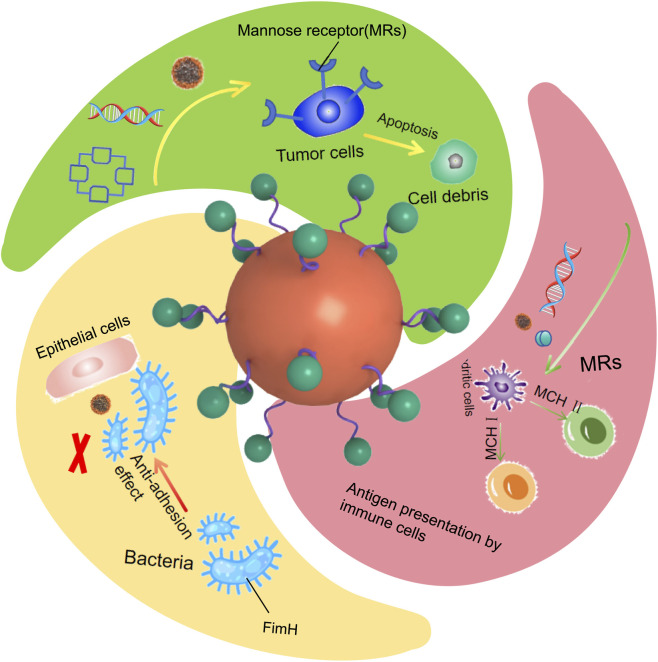
Mechanistic schematic of mannose-functionalized nanomaterials for disease therapy.

Among the many targeting ligands, mannose has garnered significant attention due to its specific binding ability to the mannose receptor (MR). The mannose receptor can recognize and bind glycoproteins with terminal mannose or fucose glycosyl groups, as well as surface polysaccharides of pathogens, mediating intracellular endocytosis through its carbohydrate-recognition domain (CTLD) (Perego et al., 2011; Cummings, 2022). Therefore, drug delivery systems using mannose as a targeting ligand (i.e., mannosylated carriers) can effectively deliver drugs to antigen-presenting cells expressing this receptor, such as macrophages and dendritic cells. MRs are highly expressed on the surfaces of various cells, including antigen-presenting cells (e.g., tumor-associated macrophages (TAMs) and dendritic cells (DCs)), as well as certain tumor cells and vascular endothelial cells. (Sallusto et al., 1995; Stahl and Ezekowitz, 1998; McKenzie et al., 2007; Hu et al., 2018; Patil and Deshpande, 2020; Lopukhov et al., 2021). This distinctive expression profile renders MRs ideal “molecular beacons,” capable of guiding drug delivery systems to precisely “navigate” to tumor microenvironments or infectious foci. Mannose-functionalized nanocarriers exploit this characteristic not only to efficiently deliver drugs into target cells via receptor-mediated endocytosis but also to leverage immunomodulatory functions—such as promoting antigen presentation and regulating macrophage polarization. The mannose-targeting strategy exerts differential regulation by leveraging the distinct receptor profiles of M1 and M2 macrophages in the early tumor microenvironment. It specifically targets via M2-highly expressed CD206, then reverses M2 metabolic and phenotypic features to induce their repolarization into anti-tumor M1 macrophages through competitive inhibition of glucose metabolism, activation of NF-κB and suppression of STAT6/PI3K signaling pathways. Meanwhile, it moderately supplies energy substrates to glycolysis-dependent M1 macrophages, enhances their glycolysis and ROS production, and amplifies their pro-inflammatory, tumor-killing and effector immune cell-recruiting functions, ultimately reshaping the tumor microenvironment from immunosuppressive to pro-inflammatory and anti-tumor, and inhibiting early tumor progression. This dual action reduces toxicity and off-target effects, thereby synergistically enhancing therapeutic outcomes (Steichen et al., 2013; Chitgupi et al., 2017). This regulatory effect stems from its “synergistic” design: on one hand, the mannose moiety itself acts as a “navigation system,” ensuring the carrier efficiently accumulates in antigen-presenting cells expressing the mannose receptor (such as macrophages and dendritic cells), creating a prerequisite for immune regulation. Although evidence suggests that mannose binding to the receptor can transmit basic immune signals, the deep reprogramming of the tumor immune microenvironment primarily relies on the active agents it delivers (Wang et al., 2020). On the other hand, and most critically, the active therapeutic agents encapsulated in the nanocarrier (such as immunomodulatory drugs, antigens, or nucleic acids) are precisely delivered and released into the target cells, directly executing “instructions.” Through mechanisms such as activating antigen presentation, promoting cytokine secretion, or reprogramming macrophage polarization, they stimulate and enhance the host’s anti-tumor immune response (Sui et al., 2020). Therefore, the immunomodulatory efficacy is the result of the combined and mutually reinforcing actions of mannose’s targeting guidance and the core functions of the loaded active agents. Currently, mannose-functionalized nanosystems based on diverse platforms—including liposomes, polymers, and inorganic nanomaterials—have been extensively developed. These systems demonstrate substantial clinical translation potential when integrated with various therapeutic modalities such as chemotherapy, photodynamic therapy (PDT), immunotherapy, gene therapy, and anti-infective therapy (Figure 1) (Patil and Deshpande, 2020).

This review aims to systematically summarize recent advances in mannose-functionalized nanocarriers for disease treatment. We intend to provide valuable insights for researchers in related fields and discuss future directions and challenges.

## 2 Structure and function of C-type lectin receptors

The biological foundation of mannose-targeted strategies lies in their high-affinity binding to specific carbohydrate-recognition proteins on the surface of target cells. These proteins primarily belong to the C-type lectin receptor (CLR) family, a class of pattern recognition receptors (PRRs) that recognize and bind specific carbohydrates in a Ca^2+^-dependent manner. Through their conserved carbohydrate-recognition domains (CTLDs), CLRs identify endogenous glycoproteins or pathogen-associated molecular patterns (Martinez-Pomares et al., 2001; Hu et al., 2018; Nasu et al., 2020), playing pivotal roles in maintaining organismal homeostasis and initiating immune responses (Taylor et al., 2005; Zhang et al., 2011; Ohnishi et al., 2012; Sorvillo et al., 2012; Goyal et al., 2016; Ma et al., 2019; Raymond et al., 2020).

CLRs can be categorized into transmembrane CLRs (TM-CLRs) and soluble CLRs. Based on molecular structure, TM-CLRs are further classified into type I TM-CLRs, type II TM-CLRs, and transmembrane C-type lectin-like receptors (TM-CTLRs) (Leur, 2014). Type I TM-CLRs include the mannose receptor (MR) and ENDO180 (mannose receptor C type 2) (Hu et al., 2018). It is noteworthy that although ENDO180 is named “mannose receptor C type 2,” it is not an ideal or direct target for mannosylated carrier targeting. ENDO180 is a collagen endocytic receptor, and its core biological function is to recognize and internalize collagen in the extracellular matrix rather than mannose, playing a critical role in tissue remodeling and repair (Zhao B. et al., 2022; Iwahashi et al., 2025). Type II TM-CLRs encompass dendritic cell-specific intercellular adhesion molecule-3-grabbing non-integrin (DC-SIGN), Langerin (CD207), and macrophage galactose-type lectin (MGL) (Andrade et al., 2020).

## 3 The mannose receptor

**FIGURE 2:**
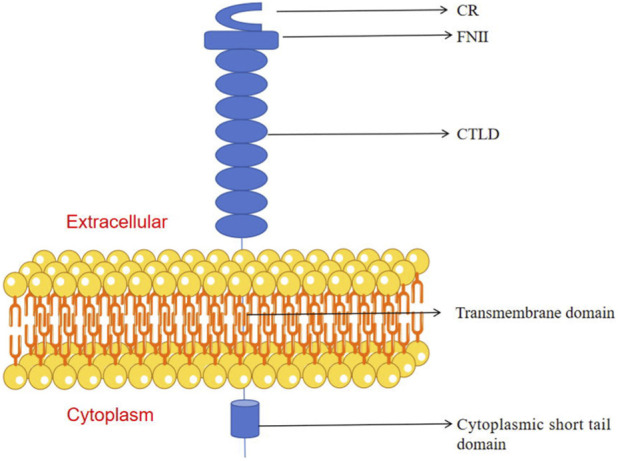
Schematic representation of the mannose receptor structure: comprising a cysteine-rich (CR) domain, fibronectin type II (FNII) domain, eight tandem C-type lectin-like domains (CTLDs), a transmembrane domain, and a short cytoplasmic tail domain.

The mannose receptor (MR) is a prototypical member of the CLR superfamily, expressed in both mice and humans with mannose-binding capability, rendering it one of the primary targets for mannose-functionalized nanocarriers (Patil and Deshpande, 2020). As a type I transmembrane glycoprotein, MR possesses a unique extracellular architecture: an N-terminal cysteine-rich domain (CR), followed by a fibronectin type II domain (FNII), eight tandem carbohydrate-recognition domains (CTLD1-8), a transmembrane domain, and a short cytoplasmic tail (Figure 2) (Stahl and Ezekowitz, 1998; Napper et al., 2001; Huang et al., 2007; Rajaram et al., 2017; Dalle et al., 2018; Fiani et al., 2020). CTLDs 4-8 constitute the key functional domains for recognizing and binding carbohydrate ligands such as mannose, fucose, and N-acetylglucosamine. The CR domain binds sulfated glycoproteins (e.g., pituitary hormones), while the FNII domain interacts with extracellular matrix components like collagen. This multi-domain configuration endows MR with broad ligand specificity (Vanderwall et al., 2018; Cummings, 2022).

Extensive studies in mice demonstrate widespread MR distribution, primarily on macrophages across nearly all tissues—including hepatic Kupffer cells, alveolar macrophages, and peritoneal macrophages—as well as on immature dendritic cells (imDCs) (Stahl and Ezekowitz, 1998; McKenzie et al., 2007; Xu et al., 2010; Kelly et al., 2011). Additionally, In addition to tumor-associated macrophages (TAMs) and antigen-presenting cells (APCs), the mannose receptor (MR) is constitutively expressed on hepatic sinusoidal endothelial cells, splenic red pulp macrophages, lymphatic endothelial cells, and tissue-resident macrophages in normal tissues (Patten and Shetty, 2018; Uehara et al., 2022). This widespread expression implies that mannosylated nanocarriers are highly susceptible to sequestration by organs such as the liver and spleen *in vivo*, leading to enhanced first-pass clearance and reduced tumor-targeting doses. Particularly in the liver, high MR expression may cause non-specific accumulation of drugs in both hepatocytes and non-parenchymal cells, impairing therapeutic efficacy and increasing the risk of hepatotoxicity. Furthermore, MR expression exhibits distinct tumor type dependency: it is highly expressed in breast cancer, glioblastoma, and certain sarcomas, while relatively low in colorectal cancer and prostate cancer. Meanwhile, interpatient heterogeneity in MR expression is significant, influenced by the tumor microenvironment, inflammatory status, and genetic background. Therefore, systematic evaluation of MR expression profiles is imperative prior to clinical translation, and strategies such as dose optimization, combination targeting, or patient stratification should be considered to reduce off-target risks and improve therapeutic precision.

It is noteworthy that within the tumor microenvironment (TME), macrophages are polarized into an M2-like phenotype under the influence of factors such as IL-4, IL-13, and M-CSF (Xu et al., 2022). These pro-tumoral M2-type tumor-associated macrophages (TAMs) accumulate in large numbers within tumors, and their surface expression of the mannose receptor (MR) is significantly upregulated, often exceeding the levels found in resting M0-type macrophages (Fiani et al., 2020; Kramer et al., 2022). This upregulation of MR is closely associated with their functions in recognizing and internalizing mannosylated antigens and modulating immune responses. Through MR-mediated endocytosis, TAMs can uptake mannosylated self-antigens or pathogen-derived components, thereby activating downstream signaling pathways. This process promotes the secretion of immunosuppressive factors such as IL-10 and TGF-β, and enhances the expression of Arginase-1 (Arg-1), collectively fostering tumor immunosuppression, angiogenesis, and stromal remodeling, thereby accelerating tumor progression (Sutera et al., 2025).

The accumulation of TAMs within tumors and their high MR expression form a critical biological basis for utilizing mannosylated nanocarriers in targeted cancer therapy. Leveraging the MR-mediated endocytic pathway, mannosylated carriers enable the precise delivery of anticancer drugs. On one hand, they can selectively transport therapeutic agents (such as chemotherapeutic drugs or immunomodulators) into TAMs. On the other hand, the carriers themselves or their cargo can interfere with MR-related signaling pathways, modulating macrophage polarization—for instance, shifting pro-tumoral M2-type macrophages toward anti-tumoral M1-type macrophages (Zhao Z. et al., 2022). This reprogramming reverses the immunosuppressive microenvironment and enhances overall antitumor efficacy. Therefore, mannosylated nanocarriers targeting MR on TAMs represent a promising strategy for improving tumor targeting and remodeling the TME (Kramer et al., 2022; Kang et al., 2023).

In addition to efficiently mediating endocytosis for the delivery of nanocarriers, the binding of the mannose receptor (MR) to its ligands (such as mannosylated carriers) can itself trigger downstream signal transduction events, which may potentially influence cellular behavior, although the complete signaling pathway has not yet been fully elucidated (Murai et al., 1995). Currently, it is known that the intracellular segment of MR lacks classical signaling motifs, and its signal transduction primarily relies on interaction with membrane-associated immunoreceptor tyrosine-based activation motif (ITAM)-bearing adaptor proteins (such as the FcRγ chain). Upon ligand binding and clustering of MR, its intracellular domain recruits Syk family kinases, thereby initiating a series of signaling cascades. These signaling events may include: Activation of immunomodulatory signals: In antigen-presenting cells (such as dendritic cells), MR-mediated signaling can influence key pathways such as NF-κB, thereby modulating the balance between pro-inflammatory and anti-inflammatory cytokines. This directly affects the initiation or suppression of immune responses. Regulation of cell survival and polarization: In macrophages, MR signaling has been linked to the regulation of cell polarization. Studies suggest that sustained MR ligand stimulation may participate in maintaining an M2-like phenotype, promoting cell survival, or suppressing excessive inflammation by regulating pathways such as ERK/PI3K. Synergistic determination of endocytic fate: MR-triggered signaling events may collaborate with the endosomal maturation process, influencing the intracellular trafficking and ultimate fate (e.g., antigen presentation or degradation) of internalized cargo, including nanocarriers.

MR orchestrates diverse functions, serving as a pivotal bridge between homeostasis and innate/adaptive immunity. As an atypical pattern recognition receptor (PRR) or “molecular scavenger,” it binds both endogenous molecules and pathogens. First, MR recognizes endogenous ligands via its extracellular domains, facilitating their internalization and degradation to maintain homeostasis (Taylor et al., 2005; Zhang et al., 2011; Ohnishi et al., 2012; Sorvillo et al., 2012; Zhang et al., 2022; Xue et al., 2023). Specific functions include: CTLD-mediated clearance of circulating lysosomal hydrolases and myeloperoxidase, removal of senescent/necrotic cellular debris; CR-dependent regulation of pituitary prohormones; and FNII-mediated collagen clearance modulating cell-matrix adhesion. Second, MR contributes significantly to xenobiotic clearance (Gage et al., 2011). As a major phagocytic receptor, it mediates actin-dependent phagocytosis (Kerrigan and Brown, 2009) and pathogen internalization, playing crucial roles in antimicrobial defense. Reduced MR expression (Chakraborty et al., 2001) or deficiency (Kuo et al., 2002) increases host susceptibility to pathogens. Conversely, some pathogens exploit MR to evade immune responses (Kang et al., 2005) or invade host cells (Garrido et al., 2011; Macedo-Ramos et al., 2011).

MRs are particularly expressed on immature and mature dendritic cells (DCs), internalizing pathogens for presentation via major histocompatibility complex (MHC) class I and II molecules (Van Kooyk, 2008). During early endosomal processing, internalized antigens can dissociate and undergo cross-presentation on MHC class I to activate CD8^+^ T cells (Gazi and Martinez-Pomares, 2009). Soluble protein antigens bound by MRs can be routed to late endosomes/lysosomes for proteolysis (Sorvillo et al., 2012; Zheng, 2012), generating peptides presented on MHC class II to activate CD4^+^ T cells. By enhancing MHC class II presentation of pathogen-derived antigens to T cells, MR critically shapes adaptive immunity (Nizet et al., 2017). Beyond phagocytosis and antigen presentation, MR engagement triggers intracellular responses including cytokine secretion, lysosomal enzyme release, and modulation of other surface receptors (Sorvillo et al., 2012; Zheng, 2012). Recognition of microbial carbohydrates by MRs enhances macrophage uptake of bacteria, yeasts, and parasites, bolstering innate defense against diverse pathogens. Following endosomal/phagosomal acidification, ligands dissociate from receptors, which recycle to the cell surface (Huang et al., 2007; Gupta et al., 2012). MR’s role in innate immunity is well-documented for clinically significant pathogens including *Mycobacterium tuberculosis* and *Pneumocystis carinii*.

It is crucial to clearly distinguish that the core role of mannose modification in immunotherapy is to achieve targeted delivery rather than acting directly as an immunomodulator. Extensive literature indicates that mannose itself does not possess significant or predictable immunomodulatory activity at physiological concentrations (such as directly driving the polarization of macrophages from the M2 to M1 phenotype or enhancing antigen presentation). Its function is more appropriately defined as a “precision key” that can efficiently unlock the endocytic gateway of antigen-presenting cells (APCs) with high mannose receptor (MR) expression, such as certain macrophage and dendritic cell subsets (van der Zande et al., 2021; Zhang et al., 2021; Nan et al., 2022). Therefore, any observed downstream immune effects—whether the reprogramming of macrophage phenotypes, the secretion of pro-inflammatory cytokines, or the enhancement of antigen presentation capacity—essentially depend primarily on the biological activity of the delivered “cargo.” For instance, mannosylated nanoparticles loaded with rifampicin (Rif@FAM NPs) can regulate macrophage polarization (M1/M2) and enhance innate immunity against intracellular pathogens (Feng et al., 2022), while similar systems loaded with nucleic acids may mainly promote the maturation of dendritic cells and upregulate T cell proliferation (Zhang W. et al., 2018). Clarifying this causal relationship is critical for the rational design of delivery systems, accurate interpretation of their mechanisms of action, and prediction of their therapeutic potential.

The complex biological functions of the Mannose Receptor (MR) provide the fundamental rationale for using mannose as a targeting ligand. By functionalizing the surface of nanocarriers with mannose or its oligosaccharides, these mannosylated carriers are no longer perceived by the immune system as inert particles. Instead, they can be specifically and recognized with high affinity by the carbohydrate recognition domains (CTLDs 4–8) of the MR (Choi et al., 2024). This biomimetic strategy enables the nanocarriers to efficiently enter MR-positive cells through receptor-mediated endocytosis. Consequently, the non-specific passive accumulation of drugs, primarily governed by the EPR effect, is enhanced by this active, cell-specific targeting. This ligand-receptor interaction serves as the cornerstone of the targeting strategy, ensuring the precise delivery of therapeutic agents to key cells (such as TAMs, APCs, and certain tumor cells). It is this precise molecular recognition that enables the various mannosylated nanocarrier platforms discussed subsequently to achieve enhanced therapeutic efficacy in the treatment of various diseases. A summary of the mechanisms for different types of nanocarriers can be found in Table 1.

**TABLE 1:** Summary of mechanisms and therapeutic effects of different types of nanocarriers.

Type	Name	Payload	Targeting mechanism	Therapeutic effect	Ref.
Polymer nanoparticles	FIS-PM NPs	Fisetin	Target the mannose receptors of cancer cells	Anti-breast cancer effec	Yadav et al. (2024)
Peptide copolymer nanoparticles	PM/BDPI	BDPI (iodinated BODIPY, photosensitizer)	pH-responsive, acidic release of mannose for targeting tumor cells	Synergistic photodynamic and metabolic therapy	Yuan et al. (2019)
Lipid nanoparticles	Man-CUR SLNs	Curcumin	Targeting the mannose receptors on the surface of lung cancer cells and macrophages	Multi-pathway anti-tumor effect; inhibits tumor proliferation	Chae et al. (2024)
Mannitol-glycated chitosan nanoparticles	CS-NC	Antibiotics	Targeting the mannose receptor on the surface of macrophages	Enhances antibiotic activity and modulates the immune response	Coya et al. (2019)
​	Rif@FAM NPs	Rifampicin	Intracellular uptake by macrophages via mannose receptors	Remodels innate immune response, enhances anti-mycobacterial activity, and upregulates M1/M2 macrophage polarization	Feng et al. (2022)
​	PM-MCS-elex-NPS	Balornycin	Targets mannose receptors on the macrophage surface	Significantly enhanced *in vitro* anti-leishmanial activity	Esfandiari et al. (2019)
​	MCS/pGRP	PGRD DNA vaccine	Targets mannose receptors on the macrophage surface	Intranasal administration induces significantly higher and more durable anti-GRP IgG antibody titers; the nanovaccine effectively inhibits tumor cell growth	Yao et al. (2013)
​	MTC-miR1466	miR-146b mimic	Targets mannose receptors on intestinal macrophages	Immune regulation and promotion of mucosal repair	Deng et al. (2019)
​	TACTIC	Chitosan adhesion	Tumor-adhesive. It is achieved through chitosan linkage, aiming to enhance intratumoral retention rather than the traditional active targeting ligand	1. Enhance the immunogenicity of tumor cells; 2. Stimulate the pro-inflammatory response of various immune cells; 3. Reshape the tumor microenvironment after radiotherapy; 4. Trigger a systemic immune response with memory effects	He et al. (2025)
​	1. RCNP_DOX_ 2. MCNPR_848_	1. Doxorubicin 2. Requintimod	1. R6RGD-CMβCD: Targeting tumor cells through RGD peptides. 2. M2pep-cmcs: Targeting M2-type tumor-associated macrophages through M2pep peptides	1. Synergistic cytotoxicity. 2. Promote the expression of cleaved Caspase-3 and induce apoptosis. 3. Effective reprogramming of TAMs phenotypes (immune regulation). 4. It demonstrates excellent anti-cancer effects in tumor-bearing mice and is an effective combination of chemotherapy (DOX) and immunotherapy (R848)	Chen et al. (2023)
Mannose-modified nanoconjugates	hPG-Ps	Zinc porphyrin photosensitizer	Targets cellular mannose receptors	Condition-dependent antibacterial phototoxicity	Staegemann et al. (2017)
Inorganic nanocarrier	GO-PEG-Man	Rifampicin	Targets mannose receptors on the macrophage surface	Compared to the free drug, significantly enhances killing efficacy against intracellular M. bovis BCG and *M. tuberculosis* *in vitro* and *ex vivo*	Pi et al. (2019)
Self-assembled nanoparticles	MACA@ICG	Active drug (cinnamaldehyde) Photosensitizer (indocyanine green)	Mannose units on the nanoparticles bind to mannose residues on the surface of infectious microbes or mammalian cells. Responds to the acidic microenvironment of bacteria, releasing free cinnamaldehyde	Synergistic therapy: The released cinnamaldehyde acts synergistically with ICG-mediated photodynamic therapy (PDT) and photothermal therapy (PTT)	Li et al. (2024)
​	MAN-PEG-Ce6	Chlorin e6 (Ce6)	Mannosamine units on the nanoparticle surface specifically recognize and bind to overexpressed mannose receptors on macrophages	PDT effect: Upon laser irradiation, significantly increases singlet oxygen generation Anti-inflammatory efficacy: PDT effectively reduces mannose receptor-positive macrophages in plaques, exhibiting a significant anti-inflammatory effect and rapidly reducing inflammation in atherosclerotic plaques	Lee et al. (2024)
​	MCMC/HA	CpG oligodeoxynucleotides (ODNs	Dual targeting: 1. Mannosylated chitosan targets mannose receptors on macrophages 2. Hyaluronic acid (HA) targets overexpressed CD44 receptors on macrophages and certain tumor cells	Remodels the immune microenvironment and induces tumor cell apoptosis	He et al. (2017)
Antibody-polysaccharide conjugates	AGC	β-glucan	Active targeted delivery is achieved by specifically targeting tumor cells/tumor microenvironments expressing PD-L1 with anti-PD-L1 antibodies	1. It has a powerful tumor suppression effect (with a tumor inhibition rate of 86.7% in the MC38 tumor-bearing mouse model). 2. Enhance the efficacy of ICB: Promote the interaction between tumor cells and dendritic cells, and enhance the benefits of immunotherapy. 3. Reshaping the tumor immune microenvironment: Inducing an earlier immune response, promoting DC infiltration, and activating the local proliferation of pre-stored T cells within the tumor. 4. Improving immune checkpoint blockade therapy has promising prospects for clinical transformation	Wang et al. (2024)

## 4 Design strategies and innovative applications of mannose-modified materials

Mannose, as an essential monosaccharide molecule, has garnered significant research interest in the field of material modification due to its unique biocompatibility and targeted recognition capabilities. Through rational molecular design, mannose-modified materials demonstrate considerable potential in biomedical applications.

### 4.1 Biomimetic design strategy

The design of mannose-modified materials often draws inspiration from natural biomolecular structures. For instance (Jiang et al., 2022), tmimicking the structure and immunomodulatory functions of mannose-capped arabinomanannan (ManLAM), researchers have developed mannose-coated spherical lysine dendrimers (MGLD). This biomimetic approach enables precise molecular control, resulting in materials with sizes ranging from 50 to 200 nm, spherical morphology, and a positive surface charge, which are ideal for cellular interactions. The core advantage of this strategy lies in its ability to emulate natural ligand-receptor recognition mechanisms. Since mannose receptors (MR) are highly expressed on immune cells (e.g., macrophages), mannose-modified materials can specifically target these cells to regulate their biological behavior. Studies indicate that mouse bone marrow-derived macrophages co-cultured with MGLD acquire an anti-inflammatory M2 phenotype, characterized by significant MR clustering and elongated cell morphology.

### 4.2 Surface modification and functionalization strategy

Another critical design approach involves surface modification and functionalization. For example (Piasek et al., 2025), mannose-modified ZnO particles have been developed by optimizing the mannose-to-ZnO molar ratio (optimal at 0.02), effectively controlling the release rate of Zn^2+^ ions. This design maintains Zn^2+^ concentrations at a low level of 1.92–2.35 mg/mL after 1 hour, significantly outperforming unmodified samples. Key advantages of this strategy include enhanced targeting capability, controlled release behavior, and improved biocompatibility.

### 4.3 Multifunctional integrated design

Advanced mannose-modified materials employ multifunctional integration strategies. A notable example is the novel nanosystem Zn-MOF@Man/LRB (Ma et al., 2025), which achieves cascade “gut-bacteria” targeting through a sophisticated multi-layer structure: Core: A zinc-based metal-organic framework (Zn-MOF) with excellent antibacterial and anti-biofilm activity. Intermediate Layer: Surface-modified mannose (Man) specifically recognizes and binds to FimH pili on *E. coli*, inducing pathogen aggregation. Outer Layer: A *Lactobacillus reuteribiofilm* (LRB) protects the nanoparticles from degradation in the acidic gastric environment while conferring intestinal targeting and immunomodulatory functions. This design overcomes limitations of conventional nanodrugs, such as gastric acid degradation and poor targeting. Upon reaching the intestine, the system sequentially enables accumulation at inflammatory sites, precise clearance of *E. coli*, promotion of intestinal stem cell differentiation, barrier repair, and microbiota balance restoration. Consistent efficacy in anti-diarrheal and anti-inflammatory outcomes has been validated in models including mice, piglets, and human diarrheal cases. The various action links of mannosylated nanosystems are shown in Table 2.

**TABLE 2:** Action links and determinants of mannosylated nanosystems.

Action link	Core process	Main determinants	Examples
Cell Targeting and Uptake	Specific recognition and endocytosis of MR	Mannose ligand density, nanoparticle surface chemistry	Mannose modification significantly improves uptake efficiency in tumor cells (Jain et al., 2010)
Endosomal Routing and Escape	Endosomal transport, acidification, and cargo intracellular release	Nanomaterial properties (e.g., pH sensitivity), carrier structure	Li et al. designed a pH-responsive carrier to effectively penetrate and eliminate persistent bacterial biofilms (Li et al., 2024)
Downstream Immune Effects	Macrophage polarization, antigen presentation, cytokine secretion	secretionIdentity and activity of the cargo (drug)	The loaded core micelle MPC is the direct cause of inducing M1 polarization (Meng W. et al., 2024)

### 4.4 Effects of particle size, ligand density, and nanocarrier type

To achieve effective mannose-targeted delivery, the intrinsic properties and action mechanisms of nanocarriers must be systematically considered. Regarding particle size, nanoparticles smaller than 50 nm are generally believed to penetrate blood vessels more easily and be efficiently endocytosed by target cells, whereas those larger than 200 nm are more likely to be sequestered by the mononuclear phagocyte system (MPS), thereby impairing targeted accumulation at tumor sites (Meng S. et al., 2024). In terms of the multivalent effect, moderately increasing the mannose ligand density (typically 30–100 ligands per particle is recommended) can significantly enhance binding affinity to mannose receptors (MR) and internalization efficiency. However, excessive modification (>150 ligands per particle) may alter the surface properties of nanocarriers, conversely compromising *in vivo* stability and biodistribution. Differences in nanocarrier types are equally critical: Liposomes exhibit high biocompatibility but limited drug-loading capacity, and mannose modification on their surface is susceptible to phospholipid exchange; Polymer-based nanocarriers facilitate precise ligand modification and controlled release but require attention to the immunogenicity of their degradation products; Inorganic nanocarriers possess high drug-loading capacity and stability but suffer from poor biodegradability, with long-term safety yet to be verified. Therefore, nanocarrier design must strike a balance between targeting efficiency, drug-loading capacity, and biosafety.

### 4.5 Avoiding common experimental pitfalls

When evaluating mannose-targeting efficacy, common experimental pitfalls must be cautiously avoided. Overreliance on *in vitro* cellular uptake assays tends to overestimate targeting efficiency, as *in vitro* models fail to simulate complex *in vivo* biological barriers, protein corona formation, and non-target cell competition. It is recommended that the following control experiments be indispensable: (1) a free mannose competitive inhibition group; (2) an MR knockdown or blocking antibody treatment group; (3) a ligand-unmodified nanocarrier control group. Furthermore, most current studies remain in the proof-of-concept stage, utilizing cell lines or animal models with high MR expression, and their conclusions cannot be directly extrapolated to clinical settings. Translation-oriented research should further focus on: (1) the quantitative differences in MR expression between patients’ tumor tissues and normal tissues (especially the liver and spleen); (2) the heterogeneity of MR expression across different tumor types (e.g., triple-negative breast cancer, pancreatic cancer) and among individuals; (3) the impact of non-targeted accumulation of nanocarriers in organs such as the liver and spleen on systemic toxicity and therapeutic efficacy. Future studies are advised to integrate clinical sample analysis with imaging tracking to advance targeted strategies toward personalized therapy.

### 4.6 Pharmacokinetic and biodistribution considerations of mannosylated nanosystems in vivo

Beyond targeting efficiency and intracellular fate, the *in vivo* pharmacokinetic and biodistribution characteristics of mannosylated delivery systems are core considerations for their clinical translation. The systemic exposure of the system, as well as its accumulation in target and non-target tissues, directly determine its therapeutic efficacy and potential toxicity. On one hand, mannose modification aims to enhance the enrichment of the system in lesion tissues with high mannose receptor (MR) expression (e.g., tumors, inflammatory sites) through an active targeting mechanism. Successful cases have shown that compared with unmodified nanoparticles, mannosylated systems can achieve higher accumulation and longer retention time in these sites (Yadav et al., 2024). However, on the other hand, mannose modification may also significantly affect the composition of the plasma protein corona of the system. Lectins in plasma (such as mannose-binding lectin, MBL) or other sugar-binding proteins can interact with mannose to form a unique “sugar-related protein corona.” This protein corona alters the “biological identity” of the nanoparticles, usually accelerating their recognition and clearance by the mononuclear phagocyte system (MPS) such as the liver and spleen, leading to relatively high accumulation in the liver and spleen (Shi et al., 2017; Li S. et al., 2020).

The design of mannose-modified materials is evolving toward intelligence, precision targeting, and personalization. These innovative strategies not only expand the applications of carbohydrate-based modifications but also provide new solutions for drug delivery, tissue engineering, and immunoregulation. With advancements in nanotechnology, synthetic biology, and artificial intelligence, the development of mannose-modified materials is expected to become more precise and efficient, driving breakthroughs in biomedicine.

## 5 Comparative analysis of mannose-targeting strategies: relative advantages and clinical limitations

While mannose demonstrates distinct targeting potential, a comparative analysis within the broader spectrum of targeting ligands is essential to objectively evaluate its relative advantages and challenges. This section systematically compares mannose with commonly used ligands in the domains of oncology and infectious disease therapy.

### 5.1 Comparison with tumor-targeting ligands

Compared to folic acid—widely employed in tumor targeting—mannose exhibits unique advantages in immunotherapy due to its ability to target antigen-presenting cells (e.g., dendritic cells and macrophages), thereby avoiding off-target effects caused by folate receptor expression in normal tissues (Mirzanejad et al., 2025). In terms of sensitivity (affinity), the binding constant (Kd) of mannose to its receptors typically falls within the micromolar range. Although this is lower than the nanomolar-level affinity of high-affinity ligands (e.g., RGD peptides targeting integrin αvβ3), this moderate affinity facilitates drug-receptor complex internalization and recycling, preventing surface “stagnation” due to excessively strong binding. However, regarding selectivity, RGD peptides exhibit relatively limited specificity as integrins are broadly expressed in neovascularure and various tumor cells. In contrast, mannose targets tumor-associated macrophages (TAMs), offering a unique pathway for modulating the tumor microenvironment.

### 5.2 Comparison with ligands for infectious disease therapy

In infectious disease therapy, mannose-based strategies complement monoclonal antibodies (mAbs) and host cell receptor ligands (e.g., CD4 mimetics). Monoclonal antibodies (e.g., anti-HIV-1 gp120 antibodies) typically exhibit high sensitivity (picomolar to nanomolar affinity) and strain specificity, but their development is costly, and viral mutations can lead to escape mechanisms (Peng J. et al., 2022; Lima et al., 2025). In comparison, mannose targets relatively conserved myeloid cell receptors (e.g., mannose receptors, MR) on the host side, providing broader activity spectra and lower production costs. However, its affinity is significantly lower than that of antibodies, potentially resulting in reduced efficacy under low antigen load conditions. Additionally, when compared to cationic cell-penetrating peptides (e.g., TAT peptide), which enter most cells via non-specific electrostatic interactions (Karimi et al., 2017), mannose relies on receptor-mediated endocytosis. While this grants superior cellular selectivity, it often comes at the cost of lower internalization efficiency.

### 5.3 Comparison with other sugar-based ligands

Relative to galactose—a ligand for the asialoglycoprotein receptor (ASGPR) used in liver targeting—both leverage natural glycan recognition pathways. ASGPR expression is highly specific to hepatocytes (Sun et al., 2024), whereas mannose receptors are more widely distributed. Consequently, while galactose excels in liver parenchymal targeting, mannose holds advantages in targeting hepatic immune cells (e.g., Kupffer cells) for intracellular pathogen clearance (e.g., Leishmaniaor viruses). Clinically, galactose faces challenges from endogenous competitive inhibition, whereas mannose, though less affected by such competition, is more susceptible to variability in individual immune states.

A systematic comparison of mannose-based targeting strategies with other carbohydrate or polysaccharide ligands that possess definite immunomodulatory functions can more profoundly reveal the design logic and application boundaries of carbohydrate-mediated targeted delivery in tumor immunotherapy. Specifically, compared with β-glucan, mannose mainly targets mannose receptors on the surface of antigen-presenting cells, while β-glucan (such as the antibody-β-glucan conjugate AGC reported in the literature) can simultaneously activate pattern recognition receptors like dectin-1, exhibiting a unique “bridging” effect in enhancing the interaction between dendritic cells and tumor cells (Reid et al., 2009; Cao et al., 2023; Wang et al., 2024). The two are complementary and synergistic in immune activation pathways. Compared with chitosan and its derivatives (such as the TACTIC construct and M2pep-CMCS), mannose has higher targeting specificity. However, relying on its cationic properties and ease of functionalization, chitosan is more flexible in achieving tumor microenvironment adhesion, loading diverse immune agonists (e.g., R848), and reprogramming the phenotype of tumor-associated macrophages (Virmani et al., 2023; Xu et al., 2024). Furthermore, compared with polysaccharide ligands such as hyaluronic acid (targeting CD44) and cyclodextrin derivatives (e.g., R6RGD-CMβCD), mannose is more focused on immune cells in terms of targeted cell subsets. Nevertheless, hyaluronic acid and cyclodextrin systems exhibit unique material advantages in direct tumor cell targeting and controlled drug release (Chen et al., 2023; Luo et al., 2024).

## 6 Application of mannose-targeted nanosystems in multimodal therapy

Leveraging their precise targeting capability toward specific immune and tumor cells, mannose-functionalized nanocarriers have emerged as a versatile delivery platform. This platform enables seamless integration with diverse cutting-edge therapeutic modalities—including conventional chemotherapy, photodynamic therapy (PDT), and emerging immunotherapies or gene therapies—thereby significantly enhancing treatment efficacy while reducing adverse effects across multiple disease models (Jin et al., 2023; Liu et al., 2025; Zhou et al., 2025; Safarzadeh et al., 2025).

### 6.1 Application in cancer

Cancer, a prevalent disease threatening human health, is primarily managed through surgery, pharmacotherapy, and radiotherapy. For malignancies, the selection of antitumor agents is particularly critical. Conventional chemotherapy remains a cornerstone, yet it faces the fundamental challenge of non-specific biodistribution. This not only causes severe damage to healthy tissues (i.e., systemic toxicity) but also limits effective drug concentrations at tumor sites, resulting in a narrow therapeutic index and multidrug resistance (MDR) (Al Bostami et al., 2022). With advances in molecular medicine, novel therapies such as immunotherapy and targeted agents have demonstrated promising efficacy (Liu et al., 2025; Zhou et al., 2025). Nevertheless, the pursuit of safe, effective, and affordable antitumor drugs remains a priority in oncology research. In recent years, a growing body of transcriptomic evidence has delineated the expression characteristics of the mannose receptor (MR) in tumors. The research team led by Academician Qimin Zhan (Wang et al., 2023) analyzed spatial transcriptomics data of lung adenocarcinoma and found that CD206, as a core marker of M2-like macrophages, is co-expressed with CD68 and CD163, significantly enriched in invasive subtypes, and distributed in both tumor and stromal regions. Multiplex immunohistochemistry (mIHC) validation confirmed that CD68^+^CD163^+^CD206^+^ macrophages specifically accumulate in perivascular areas, consistent with the trend of elevated CD68 expression in The Cancer Genome Atlas (TCGA) cohort. CellPhoneDB analysis revealed that CD206^+^ macrophages participate in intercellular communication through ligand-receptor pairs such as MIF-TNFRSF14, mediating an immunosuppressive microenvironment and further supporting their functional relevance in promoting tumorigenesis. A study (Wu et al., 2023) demonstrated that in the invasive zone of hepatocellular carcinoma, single-cell RNA sequencing (scRNA-seq) data showed significant enrichment of CD206 gene expression in macrophage subsets. These macrophages are distributed in both tumor and stromal regions of the invasive zone, with transcriptional profiles associated with anti-inflammatory and pro-tumorigenic functions. Spatial transcriptomics further validated that CD206^+^ macrophages are highly aggregated in the invasive zone, adjacent to damaged hepatocytes with high expression of serum amyloid A1/A2 (SAAs), and mediate M2 polarization through the SAAs-TLR2 axis. Transcriptomic data from relevant cohorts and mouse models also confirmed the association between CD206 enrichment in high-invasive hepatocellular carcinoma subtypes and tumor progression. In a multidimensional sequencing analysis of myeloid cells in glioma (Miller et al., 2025), consensus non-negative matrix factorization (cNMF) was applied to analyze scRNA-seq data from 85 glioma samples, identifying CD206 as a core marker gene of the “scavenger immunosuppressive program.” This gene is expressed in various myeloid cells, including microglia and monocytes. Spatial transcriptomics (Visium) validation showed that CD206^+^ cells are enriched in hypoxic tumor regions and perivascular niches, highly correlated with programs related to mesenchymal transition (MES). Clinical correlation analysis indicated that high CD206 expression is significantly associated with resistance to PD-1 inhibitor therapy and poor overall survival in glioblastoma patients.

Substantial evidence indicates that mannose-functionalized nanocarriers, as active targeted delivery systems, offer an attractive strategy to address these challenges. Given the frequent overexpression of mannose receptors on cancer cells, mannose-modified nanocarriers can selectively bind to these receptors on both tumor cells and tumor-associated immune cells, enabling precise therapeutic delivery (Liu X. et al., 2023).

#### 6.1.1 Application in chemotherapy

The advantages and application of active targeting nanotechnology in the field of drug delivery provide a new approach to overcoming challenges faced by traditional chemotherapy, such as low bioavailability and poor targeting. Taking fisetin (FIS), a natural flavonoid, as an example, although it has significant anticancer activity, its extremely poor water solubility severely limits its clinical application. Mannose-modified nanocarriers offer multidimensional strategies to address the delivery issues of such hydrophobic drugs. Studies have shown (Vedove et al., 2018) that mannose modification can enhance chemotherapy efficacy through the following mechanisms: (i) achieving efficient targeted recognition by leveraging the high expression of mannose receptors on the surface of tumor cells; (ii) improving the *in vivo* distribution characteristics of drugs and increasing drug accumulation at tumor sites; (iii) enhancing the selective uptake of drugs by cells. These properties work together to significantly improve the bioavailability and therapeutic effects of hydrophobic drugs. Studies by Wang et al. (2019) have confirmed that mannose-based nanodelivery systems exhibit broad prospects in tumor therapy due to their excellent performance. This strategy, which combines active targeting and enhanced delivery, provides an important direction for the development of high-efficiency and low-toxicity cancer chemotherapy regimens.

Similarly, to overcome the poor aqueous solubility and bioavailability of fisetin (FIS)—a natural flavonoid with anticancer, antioxidant, and neuroprotective properties—Yadav et al. (2024) developed polymannose-conjugated nanoparticles (FIS-PM NPs). This design not only enhanced FIS solubility but also promoted its accumulation in breast cancer cells through mannose-mediated targeting, amplifying cytotoxicity and apoptosis induction.

As excellent drug carriers, Solid Lipid Nanoparticles (SLNs) possess characteristics such as good biocompatibility, high drug-loading capacity, and ease of large-scale production, making them an ideal platform for constructing targeted delivery systems. Jain et al. (2010) successfully developed mannose-modified SLNs loaded with doxorubicin. Although doxorubicin exhibits significant antitumor efficacy, its clinical application is limited due to a narrow therapeutic window and severe toxic side effects. Experimental results showed that mannosylated SLNs have favorable physicochemical properties: their *in vitro* release follows a biphasic pattern (initial rapid release followed by slow release), and they have low hemolytic toxicity. In the A549 lung cancer cell model, mannosylated SLNs demonstrated the strongest cytotoxicity and the highest cellular uptake rate, which is mainly attributed to mannose receptor-mediated endocytosis. Further *in vivo* studies confirmed that mannose modification significantly improved the pharmacokinetic properties of doxorubicin, prolonged the drug half-life, and enhanced bioavailability. More importantly, this delivery system enabled higher concentrations of drug accumulation in tumor tissues while significantly reducing damage to liver and kidney tissues. In summary, mannose-modified SLNs achieve precise delivery of antitumor drugs through a receptor-mediated targeting mechanism, providing an effective strategy to overcome the toxic side effects of traditional chemotherapy and showing broad application prospects in the field of cancer therapy.

Chrysin, a natural flavonoid, has significant antitumor activity, but its low oral bioavailability severely limits its clinical application. To improve its targeted delivery efficiency, Pandey et al. (2021) developed mannose-modified chrysin-loaded solid lipid nanoparticles (MC-SLNs) and systematically evaluated their potential for targeted therapy of gastric cancer. In the study, chrysin-loaded SLNs (C-SLNs) were prepared via high-speed shear emulsification technology; the formulation was optimized using a three-factor, two-level full experimental design, and characterized by Fourier transform infrared spectroscopy (FTIR), differential scanning calorimetry (DSC), X-ray diffraction (XRD), and scanning electron microscopy (SEM). The results showed that the optimized C-SLNs had a particle size of 285.65 nm with uniform distribution, an encapsulation efficiency of 74.43%, and a drug release rate of 64.83%. After mannose modification, the particle size of MC-SLNs increased to 307.1 nm, the encapsulation efficiency remained at 70.8%, and the drug release rate was adjusted to 62.87%. *In vitro* biological evaluation indicated that the hemolytic toxicity of MC-SLNs was significantly lower than that of free chrysin and unmodified C-SLNs, showing good hemocompatibility. At the cellular level, MC-SLNs exhibited the strongest cytotoxicity against gastric cancer cells (AGS) and were preferentially taken up by cancer cells—this property is attributed to the mannose receptor-mediated targeted recognition mechanism. This study confirmed that mannose modification not only improves the biocompatibility of SLNs but also enhances the killing effect of chrysin on gastric cancer cells through specific targeting. As a novel targeted delivery system, MC-SLNs provide an effective strategy to overcome the low bioavailability of chrysin and show promising application prospects in the field of targeted gastric cancer therapy.

In conclusion, mannose-modified nanocarriers exhibit significant advantages in improving drug delivery efficiency. Whether for enhancing the bioavailability of natural flavonoids (e.g., fisetin, chrysin) or improving the targeting of traditional chemotherapeutic drugs (e.g., doxorubicin), mannose modification enables precise tumor targeting through receptor-mediated endocytosis. Studies have confirmed that this strategy not only significantly increases drug accumulation in tumor tissues and enhances anticancer activity but also effectively reduces systemic toxicity and improves pharmacokinetic properties. These advantages make mannose-modified nanocarriers an effective means to overcome the limitations of traditional chemotherapy, provide an important direction for the development of high-efficiency and low-toxicity tumor treatment regimens, and hold broad clinical application prospects in the field of precision medicine.

#### 6.1.2 Applications in photodynamic therapy: enhancing targeted accumulation of photosensitizers

**FIGURE 3:**
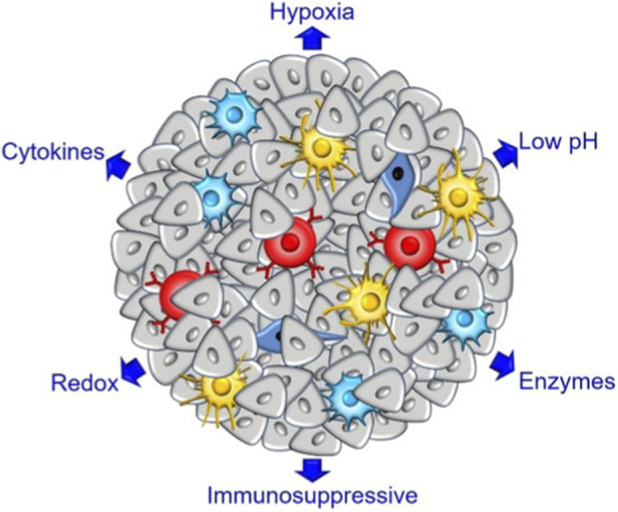
Schematic representation of the tumor microenvironment in solid tumors. TME refers to the non-cancerous cells and components presented in the tumor, including blood vessels, extracellular matrix (ECM), fibroblasts, the surrounding immune cells, molecules produced and released by them Reproduced under terms of the CC-BY license. Copyright 2022, Lei Chen et al., published by Front Bioeng Biotechno.

Photodynamic therapy (PDT) and photothermal therapy (PTT), as emerging minimally invasive techniques with spatiotemporal controllability, have demonstrated significant potential in preclinical studies (An et al., 2024; Joe et al., 2024). PDT, in particular, has garnered considerable attention due to its precise targeting capability and minimal side effects, showing clinical utility in treating malignancies, microbial infections, and vascular lesions. The therapeutic mechanism involves photosensitizers (PS) that, upon activation by specific-wavelength light, react with molecular oxygen in target tissues to generate reactive oxygen species (ROS). This process induces cell death, disrupts adjacent vasculature, and triggers immunogenic cell death (ICD) (Cramer et al., 2022).However, conventional PS agents face limitations such as poor aqueous solubility and inadequate targeting specificity, which restrict their effective accumulation at lesion sites and may cause phototoxic side effects. To overcome these challenges, researchers have integrated PS with nanomaterials through various functionalization strategies to optimize their performance (Figure 3) (Chen et al., 2022; Barik et al., 2024). Mannose-functionalized nanocarriers provide an exceptional solution to these bottlenecks. By encapsulating PS or photothermal agents, these nanosystems not only improve pharmacokinetic behavior but crucially achieve active targeting of mannose receptors (MR). This enables precise delivery to MR-overexpressing tumor cells or immune cells (e.g., tumor-associated macrophages, TAMs) within the tumor microenvironment (TME), realizing “targeting-enhanced photosensitization.” Specifically, this approach significantly increases PS concentration at disease sites, thereby amplifying cytotoxic effects under identical light irradiation doses and broadening the therapeutic window.

Zhang Q. et al. (2017) developed a novel three-armed distyryl BODIPY derivative, which was covalently conjugated with mannose units (named BTM) and could co-assemble with Tween 80 to form nanomicelles (BTM-NMs) for targeted photodynamic therapy. *In vitro* studies showed that this nanosystem could be specifically recognized and internalized by MDA-MB-231 breast cancer cells through mannose-receptor-mediated endocytosis, and preferentially accumulated in lysosomes. Under light irradiation, these nanomicelles could disassemble in cellular lysosomes and generate highly efficient singlet oxygen; the generated singlet oxygen could disrupt the lysosomal membrane structure, prompting BTM to escape from lysosomes into the cytoplasm, and finally achieve efficient and selective killing of cancer cells through the photodynamic effect. This study provides valuable insights for the design of novel targeted photodynamic therapy systems.

The targeted delivery strategy mediated by mannose-functionalized nanocarriers offers a promising solution to overcome the bottlenecks of traditional photodynamic and photothermal therapies. Such carriers not only effectively improve the water solubility and *in vivo* pharmacokinetic behavior of photosensitizers but also realize the specific accumulation of drugs in tumor cells or tumor-associated immune cells through the mannose receptor-mediated active targeting mechanism, thereby significantly enhancing the therapeutic precision and killing efficiency. For example, the BTM-NMs system developed by Patil et al. is a vivid example of this strategy—it achieves efficient and selective cancer killing at the cellular level through a series of elaborate designs, including lysosomal targeting, singlet oxygen generation, and organelle escape. In conclusion, the mannose-modified nano-platform has successfully deeply integrated the “targeting” and “photosensitization” effects, promoting the development of photodynamic therapy toward higher specificity and lower toxic side effects, and laying an important foundation for the design of the next-generation of tumor-targeted therapy systems.

#### 6.1.3 Application in immunotherapy

In cancer therapy, mannose-modified nanocarriers not only enable precise drug delivery but, more importantly, actively participate in immunomodulation by targeting key immune cells within the tumor microenvironment (TME)—such as tumor-associated macrophages (TAMs) and dendritic cells (DCs)—offering novel strategies to overcome tumor immunosuppression. Their immunomodulatory functions are primarily achieved through the following mechanisms. (i) Reprogramming Tumor-Associated Macrophages (TAMs): TAMs often exhibit a pro-tumor M2 phenotype and highly express the mannose receptor (MR). Mannosylated nanocarriers are specifically internalized into M2-type TAMs via MR-mediated endocytosis. The loaded agents (e.g., immunomodulators or chemotherapeutic drugs) can then induce their polarization towards the anti-tumor M1 phenotype (Meng W. et al., 2024), thereby enhancing phagocytic activity, promoting pro-inflammatory cytokine secretion, and improving antigen presentation capacity, ultimately reversing the immunosuppressive TME. (ii) Enhancing Antigen Presentation and T Cell Activation:Mannosylated carriers can target MRs (and DC-SIGN) on the surface of DCs, facilitating the uptake and processing of tumor antigens. Nanosystems co-delivering antigens or adjuvants enhance antigen presentation via MHC class I and II pathways, thereby activating cytotoxic CD8^+^ T cells and helper CD4^+^ T cells to initiate a specific anti-tumor immune response. (iii) Synergizing with Immune Checkpoint Blockade Therapy: Studies indicate that immunomodulators (e.g., cytokines or small-molecule inhibitors) delivered by mannosylated nanocarriers can remodel the TME and increase sensitivity to immune checkpoint inhibitors (e.g., anti-PD-1/PD-L1 antibodies). For instance, by inhibiting the function of M2-type TAMs or regulatory T cells (Tregs), mannosylated nanocarriers may reverse immune resistance and enhance the efficacy of combination therapies (Liu et al., 2024). (iv) Inducing immunogenic cell death (ICD): When loaded with chemotherapeutic agents or photosensitizers, mannosylated nanocarriers can induce ICD in target cells. This process leads to the release of tumor-associated antigens and damage-associated molecular patterns (DAMPs), such as ATP and HMGB1, which promote the recruitment of DCs and cross-presentation of antigens, thereby stimulating a systemic anti-tumor immune response.

##### 6.1.3.1 Delivery of immunomodulators

Immunomodulators, such as cytokines, oligonucleotides, and nucleic acid drugs, play a core role in the treatment of cancer, autoimmune diseases, and inflammatory diseases. However, their clinical application is often limited by systemic toxic side effects caused by non-specific distribution, insufficient bioavailability at target sites, and difficulty in effectively penetrating biological barriers to reach specific immune cell populations. Therefore, the development of targeted delivery systems capable of precisely delivering immunomodulators to target immune cells (e.g., macrophages, dendritic cells) has become a key Frontier for improving therapeutic efficacy and reducing side effects.

Mannose-functionalized nanocarriers exhibit significant advantages in the delivery of immunomodulators, especially in terms of targeting antigen-presenting cells (APCs) such as macrophages and dendritic cells.

He et al. (2017) engineered dual-targeting mannosylated carboxymethyl chitosan-hyaluronic acid/protamine sulfate/oligonucleotide nanoparticles (MCMC/HA/PS/ODN NPs) to overcome tumor-associated immunosuppression. Compared to single-targeted (HA/PS/ODN NPs, MCMC/PS/ODN NPs) and non-targeted (CMC/PS/ODN NPs) counterparts, this system demonstrated superior macrophage targeting, minimal cytotoxicity, and potent immunostimulation, highlighting CpG-ODN’s therapeutic potential. Complementarily, Deng et al. (2019) developed mannosylated trimethyl chitosan/miR-146b nanoparticles (MTC-miR-146b-NPs) for ulcerative colitis treatment. Exhibiting high encapsulation efficiency and sustained release, these NPs selectively targeted intestinal macrophages without cytotoxicity, promoting mucosal regeneration in DSS-induced colitis models and suppressing colitis-associated cancer in miR-146b−/− mice.

In conclusion, mannose-functionalized nanocarriers, with their precise targeting ability, provide a highly promising pathway for the delivery of immunomodulators. From targeting macrophages in the tumor microenvironment (TME) to reverse immunosuppression, to precisely regulating local immune homeostasis in intestinal inflammation, this strategy demonstrates the dual advantages of enhancing therapeutic efficacy and reducing off-target toxicity. In the future, with the in-depth understanding of the surface receptor profiles of immune cell subsets, mannosylated delivery systems are expected to achieve higher levels of cellular specificity, thereby promoting the application of immunotherapy in a broader range of disease fields.

##### 6.1.3.2 Applications in vaccine

With the development of vaccinology, nucleic acid vaccines (such as DNA vaccines and mRNA vaccines) have shown great application prospects due to their ability to efficiently induce cellular and humoral immunity. However, the full exertion of their efficacy heavily relies on the effective delivery of nucleic acid antigens into the interior of antigen-presenting cells (APCs) to initiate subsequent specific immune responses. This process faces multiple physiological barriers: naked nucleic acids are easily degraded rapidly *in vivo*, difficult to cross cell membranes, and unable to actively home to key immune cells. Therefore, the development of safe and efficient targeted delivery systems has become the key to breaking through the bottleneck in the field of nucleic acid vaccines.

In vaccine applications, gene therapy faces the challenge of effectively delivering nucleic acids to APCs. Naked nucleic acids undergo rapid enzymatic degradation and exhibit poor membrane permeability. Crucially, eliciting effective immune responses or achieving precise gene silencing requires targeted delivery to antigen-presenting cells (APCs), such as macrophages and dendritic cells (DCs). By leveraging the high expression of mannose receptors and DC-SIGN on the surface of APCs, mannose-functionalized nanocarriers act as “molecular navigation beacons,” significantly improving the specificity and efficiency of nucleic acid delivery (He et al., 2013). Yao et al. (2013) used mannosylated chitosan-plasmid GRP nanoparticles (MCS-pGRP NPs) as a vaccine delivery platform. This system effectively protects DNA from nuclease degradation and enhances transfection efficiency through mannose receptor (MR)-mediated endocytosis. Its performance is superior to that of unmodified chitosan carriers, providing important technical support for the development of nucleic acid vaccines.

In conclusion, mannose-functionalized nanocarriers, through their unique active targeting mechanism, provide a powerful solution to the delivery challenges of nucleic acid vaccines. They not only protect the integrity of genetic material during delivery but also, like a “precision-guided” system, directly deliver vaccine payloads into “commanders” of immune responses such as dendritic cells and macrophages, thereby efficiently initiating immune responses. This strategy not only greatly enhances the translational potential of nucleic acid vaccines but also lays a solid technical foundation for developing a new generation of efficient, safe and targeted vaccines.

#### 6.1.4 Applications in gene therapy

Gene therapy—a promising approach to treat or prevent diseases by introducing exogenous genes into target cells (Tamm et al., 2001)—faces critical challenges in delivering fragile, negatively charged nucleic acids (e.g., plasmid DNA, siRNA) to specific subcellular compartments, particularly for DNA vaccines and RNA interference (RNAi)-based strategies (Wu et al., 2020; An et al., 2024).The efficacy of gene therapy critically depends on the optimal characteristics of delivery systems: high targeting specificity, low cytotoxicity, effective protection of genetic material against nuclease degradation, and superior gene transfection efficiency. Nanocarriers modified with mannose and its derivatives, owing to their exceptional targeting capabilities, hold substantial therapeutic potential and have progressively emerged as a prominent research focus in disease treatment strategies in recent years.

Although layered double hydroxide nanoparticles (LDH NPs) show great potential as gene delivery carriers, their insufficient targeting limits their clinical application. To address this challenge, a study (Li et al., 2018) developed a mannose-modified SiO_2_-coated LDH nanocomposite (Man-SiO_2_@LDH) for the targeted delivery of small interfering RNA (siRNA). For the first time, this study conjugated mannose as a targeting molecule to the surface of the SiO_2_@LDH composite carrier. Cellular uptake experiments confirmed that compared with unmodified LDH, Man-SiO_2_@LDH could deliver siRNA to osteosarcoma (U2OS) cells more efficiently. When loaded with commercial cell death siRNA (CD-siRNA), Man-SiO_2_@LDH exhibited a stronger cancer cell-killing effect, which was attributed to the mannose receptor-mediated targeted recognition and endocytic mechanism. This study demonstrated that mannose modification, by specifically binding to the highly expressed mannose receptors on the surface of tumor cells, significantly improved the delivery efficiency and therapeutic effect of gene drugs. The Man-SiO_2_@LDH nano-platform successfully achieved the targeted delivery of siRNA to tumor cells, providing a new strategy to overcome the targeting challenges in gene therapy and holding important application value in the field of precision cancer treatment.

Dendritic cell (DC)-based immunotherapy, as a novel strategy for cancer treatment, is often limited in efficacy by negative immune regulatory molecules such as indoleamine 2,3-dioxygenase (IDO). To break through this bottleneck, a study (Zhang Y. et al., 2018) developed a novel DC-targeted siRNA delivery system—mannose-modified gold nanorod carriers (man-GNR-siIDO)—to enhance anti-tumor immune responses by specifically silencing the IDO gene. Using mannose as a guiding molecule, this nanosystem skillfully leveraged the high expression of mannose receptors on the surface of DCs to achieve precise siRNA delivery. For the first time, the study combined man-GNR-siIDO with the Flt3-L-mediated *in vivo* DC mobilization strategy for lung cancer immunotherapy. Experimental results showed that this delivery system could effectively promote DC maturation, upregulate the proliferation of tumor antigen-specific T cells, and enhance tumor-specific cytotoxicity. In the Lewis lung cancer model, the combined treatment regimen significantly inhibited tumor growth and delayed tumor formation. The significance of this study lies in three aspects: it realized targeted gene silencing of *in vivo* mobilized DCs for the first time, breaking through the technical limitations of traditional *in vitro* DC vaccine preparation; it improved the delivery efficiency of gene drugs through the mannose-mediated active targeting mechanism; and it provided a new strategy to overcome immunosuppression in the tumor microenvironment. This therapeutic model, which combines DC mobilization with targeted gene silencing, has brought a new breakthrough to the field of cancer immunotherapy and shows broad clinical application prospects.

Gene therapy has great potential in the field of disease treatment, but its development is limited by the challenge of targeted delivery of nucleic acid drugs. Mannose-modified nanocarriers, with their precise targeting properties, provide an innovative solution to break through this bottleneck. For example, in osteosarcoma treatment, mannose-modified LDH nanocomposites significantly improve the delivery efficiency of siRNA and tumor-killing effect through receptor-mediated endocytosis; in DC-based immunotherapy, mannose-guided gold nanorod systems successfully achieve targeted silencing of the IDO gene and effectively activate anti-tumor immune responses. These studies collectively confirm that mannose modification not only enhances the targeting ability of gene drugs to specific cells but also significantly improves therapeutic effects by regulating the expression of key genes. With the deepening of research, mannose-based nano-delivery systems are expected to become an important technical platform in the field of gene therapy, opening up new avenues for the precise treatment of diseases such as cancer.

#### 6.1.5 Limitations of current research

However, current studies primarily focus on demonstrating the efficacy of specific nanocarriers in particular models, lacking cross-comparisons and in-depth mechanistic investigations. Several critical issues urgently require resolution: (1) Methodological flaws in validating targeting specificity: Most studies only confirm targeting capability through increased cellular uptake, but lack key competitive inhibition experiments or receptor knockdown/blockade controls, failing to fully exclude contributions from non-specific uptake pathways. Future research must adopt more rigorous experimental designs, including pre-incubation with free mannose, use of MR-knockout cell lines, or MR-blocking antibodies, to verify receptor-dependent uptake. (2) Insufficient assessment of translational relevance: Most preclinical studies are conducted in cell lines with high MR expression or immunocompetent mouse xenograft models, which cannot fully simulate the heterogeneity of human tumors. MR expression varies drastically across different cancer types (e.g., triple-negative breast cancer vs. prostate cancer) and among individual patients. Additionally, the acidic, hypoxic, and immunosuppressive conditions in the tumor microenvironment may affect nanocarrier function and drug release. Current research generally lacks testing in patient-derived xenograft models or genetically engineered mouse models, and few studies explore nanocarrier behavior in in vitro 3D models that mimic the human tumor microenvironment. Scientific progress also stems from recognizing failures and limitations. The field needs more studies reporting negative or neutral results to clarify the technical boundaries: (1) Efficacy limitations in low MR-expression tumors: In tumor models with low MR expression, mannose-targeted strategies may not offer significant advantages. For instance, in certain prostate cancer or colorectal cancer models, the therapeutic difference between targeted and non-targeted nanocarriers may be insignificant. Honest reporting of such cases helps accurately define the indications for this technology. (2) Underestimated carrier-related toxicity: Beyond the inherent toxicity of drugs, carrier materials (e.g., certain cationic polymers or inorganic nanomaterials) may induce immune activation, oxidative stress, or long-term retention toxicity. Mannose modification may alter the *in vivo* distribution of nanocarriers but cannot necessarily eliminate the toxicity risks of the materials themselves. More comprehensive safety evaluations are required, including long-term toxicological studies and immunogenicity testing. (3) Risks of synergy and antagonism in combination therapy: When mannosylated nanocarriers are combined with radiotherapy, chemotherapy, or immune checkpoint inhibitors, unpredictable interactions may occur. For example, locally induced immune activation by nanocarriers may theoretically enhance immunotherapy but could also trigger excessive inflammatory responses. Currently, there is a lack of systematic studies to unravel the mechanisms and outcomes of these complex interactions.

### 6.2 Applications in anti-infective therapy: targeting intracellular sanctuaries and overcoming drug resistance barriers

Numerous pathogens posing severe threats to human health—including specific bacteria, fungi, and parasites—have evolved an ingenious survival strategy: they internalize into host cells (particularly immune cells like macrophages) to establish “intracellular sanctuaries.” Within these protective niches, pathogens evade host circulatory antibodies and complement attacks while resisting antibiotics that fail to penetrate cell membranes effectively, thereby forming persistent “pathogen reservoirs” for recurrent infections (Zhang and Cheng, 2022). To address this clinical challenge, mannose-functionalized nanocarriers provide an ideal “Trojan horse” strategy by exploiting the mannose receptors (an endogenous pattern recognition receptor, PRR) highly expressed on phagocytes like macrophages, which naturally recognize mannose residues on pathogen surfaces. Encapsulating antimicrobial agents within these nanocarriers hijacks this phagocytic pathway, enabling efficient active-targeted delivery to infected cells and achieving bactericidal concentrations directly within pathogen strongholds.

Cellular invasion often requires mannose-mediated interactions (Kawakami et al., 2000; Hu et al., 2016), and bacterial lectins represent key virulence mechanisms for evading human defenses. Pathogens express surface lectins (e.g., fimbrial adhesin FimH) that bind host cell glycans to induce infection. Mannose-binding lectins (MBLs)—membrane proteins with high affinity for sugar moieties (Wellens et al., 2008)—exhibit species-specific binding: *Streptococcus pyogenes* and *Staphylococcus aureus* show high MBL affinity; *Pseudomonas aeruginosa*, *Staphylococcus epidermidis*, *non-group A β-hemolytic streptococci*, and *Streptococcus pneumoniae* display weak/no affinity; while *Klebsiella spp.* and *Escherichia coli* demonstrate variable binding capacities (Heitzeneder et al., 2012).

To eradicate intracellular bacteria effectively, an ideal delivery system must achieve not only host cell targeting but also subcellular co-localization with pathogens. Addressing this, Feng et al. (2022) engineered a sophisticated cascade-targeting nanosystem (Rif@FAM NPs). This platform exhibits exceptional sequential targeting capability: mannose-modified nanoparticles encapsulating rifampicin (Rif@FAM NPs) first target macrophages via mannose receptor (MR) recognition. Post-uptake, acidic cleavage of Schiff base linkages in phagolysosomes releases mannose and exposes d-alanyl residues, triggering lysosomal escape and subsequent bacterial targeting through peptidoglycan binding. Concurrently, Rif@FAM NPs modulate macrophage polarization (M1/M2) to enhance innate immunity against intracellular pathogens.

**FIGURE 4:**
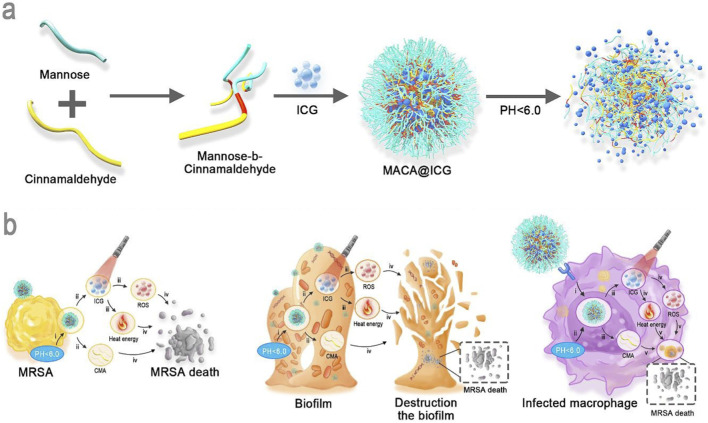
Assembly of photodynamic/photothermal pH-responsive dimeric prodrug (MACA@ICG). **(a)** Schematic illustration of MACA@ICG targeting MRSA, biofilms and infected macrophages. **(b)** Reproduced under terms of the CC-BY license. Copyright 2024, Wenting Li et al., published by Elsevier Ltd.

*Staphylococcus aureus*—a prevalent Gram-positive coccus causing community and hospital-acquired infections (Lindsay and Holden, 2004)—has developed severe resistance to first-line antibiotics like penicillin, particularly in *methicillin-resistant S. aureus* (MRSA) strains that compromise clinical anti-infective therapy (Hofer, 2019)—has developed severe resistance to first-line antibiotics like penicillin, particularly in *methicillin-resistant S. aureus* (MRSA) strains that compromise clinical anti-infective therapy (Peng L. et al., 2022) constructed a mannose-decorated pillararene-based supramolecular host-guest nanosystem (LZD–WP5*G). This system efficiently loads hydrophobic linezolid (LZD) via host-guest chemistry. MR-mediated endocytosis significantly elevates intracellular LZD concentrations, overcoming efflux pump-mediated subtherapeutic drug levels and demonstrating potent bactericidal activity against intracellular MRSA with excellent biocompatibility. Through mannose-mediated specific targeting, these nanocarriers direct diverse PS agents to pathological sites, potentiating PDT efficacy. Recently, PDT applications have expanded beyond oncology to include infectious diseases and other pathologies. In antibacterial photodynamic therapy (aPDT), mannose-functionalized nanocarriers enhance PS accumulation at infection sites, offering a potent strategy against antibiotic-resistant pathogens. Notably, *Staphylococcus aureus* exhibits high binding affinity for manno-oligosaccharides (Ikeda et al., 2007). Staegemann et al. (2017) developed a dual-responsive mannosylated hyperbranched polyglycerol multi-photosensitizer (hPG-PS) system by incorporating redox-cleavable disulfide linkers and acid-labile hydrazone bonds, which enabled targeted delivery of photosensitizers to both tumor cells and pathogenic bacteria. Li et al. (2024) developed pH-responsive dimeric prodrug nanocomposites (MACA@ICG) (Figure 4) based on mannose (MAN) and cinnamaldehyde (CMA). The system actively targets macrophages enriched in MRSA-infected areas. Within the acidic microenvironment generated by bacterial metabolism, MACA prodrugs release CMA—a broad-spectrum antibacterial agent. Combined with ICG-mediated PDT/PTT under NIR irradiation, this platform efficiently penetrates and eradicates stubborn bacterial biofilms, establishing a novel photo-controlled chemical strategy against drug-resistant infections.

**FIGURE 5:**
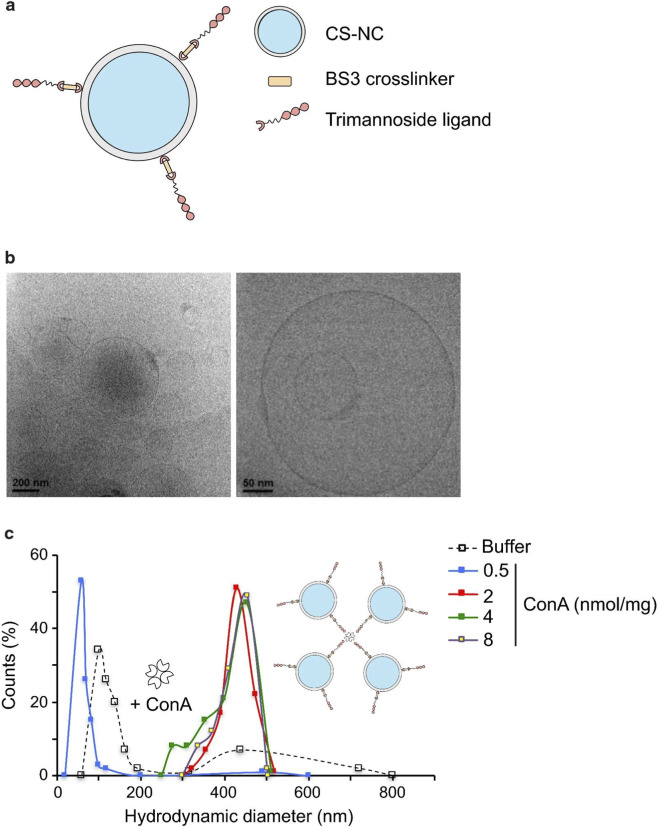
Morphological characterization of tri-mannose-grafted chitosan NCs. **(a)** Schematic representation of a mannosylated CS-NC. **(b)** CryoTEM image of CS-NCs-tri. **(c)** Hydrodynamic diameter of CS-NCs-tri incubated at various concentrations of concanavalin A, assessed by dynamic light scattering. Source: Figure 7 is reproduced from Coya JM et al. Copyright 2019, reproduced with permission from Springer Nature SharedIt.

This nanotherapeutic strategy demonstrates significant promise for chronic infectious diseases. Tuberculosis (TB), a lethal global epidemic caused by *Mycobacterium tuberculosis* (Mtb), relies on potent antibiotics (rifampicin, isoniazid, pyrazinamide, ethambutol). However, poor macrophage targeting by these drugs necessitates ≥6-month therapies, leading to hepatotoxicity (Petros et al., 2016), nephrotoxicity (Chiba et al., 2013), and drug-resistant Mtb mutants (Islam et al., 2017). Capitalizing on Mtb’s alveolar macrophage tropism, Pi et al. (2019) engineered mannose-modified graphene oxide nanovehicles (Rif@GO-PEG-MAN) for efficient rifampicin delivery This system enhanced intracellular drug accumulation and pharmacokinetics, enabling rapid endosomal escape under acidity and sustained bactericidal concentrations—potentially reducing treatment duration and side effects. Complementarily, Coya et al. (2019) utilized mannose-trimethyl chitosan nanoparticles (MTCNPs) to modulate macrophage responses during Mtb infection. RNA sequencing revealed MTCNP-induced differential expression of 900 genes, particularly those regulating oxidative phosphorylation and glucose metabolism (Figure 5). This suggests combining antimicrobials with host-directed nanocarriers represents a paradigm-shifting anti-infective strategy.

The broad applicability of this strategy extends to antiparasitic therapy. Taking leishmaniasis—a parasitic disease caused by Leishmania protozoa whose amastigote forms reside predominantly within human macrophages—as an example, current therapies rely heavily on antibiotics and other agents increasingly compromised by drug resistance (Ponte-Sucre et al., 2017). Multiple independent studies validate mannose-targeted efficacy: Afzal et al. (2019) investigated oral paromomycin (PM) for visceral leishmaniasis but confronted its low bioavailability, short half-life, and limited potency; consequently, Esfandiari et al. (2019) engineered mannosylated chitosan-dextran PM nanoparticles (MCS-dex-PM-NPs) demonstrating high macrophage targeting specificity, low cytotoxicity, and enhanced antileishmanial activity. Similarly, Afzal et al. (2019) developed mannose-thiolated chitosan/PLGA PM nanoparticles (MTC-PLGA-PM-NPs), which significantly increased macrophage uptake and parasite lethality in Leishmania donovani-infected models compared to free PM, reducing parasite loads in BALB/c mice by >80% post-treatment. Further validating this platform, Chaubey et al. (2018) synthesized mannosylated curcumin nanoformulations (Cur-MCN-NPs) that outperformed both chitosan-encapsulated and free curcumin *in vitro* and *in vivo*, achieving superior therapeutic outcomes against leishmaniasis.

Collectively, mannose-modified nanocarriers establish a robust multifunctional platform for anti-infective therapy. By enabling “targeted stealth delivery” to intracellular pathogens, they resolve the fundamental challenge of drug accessibility while concurrently overcoming resistance mechanisms, penetrating biofilms, and modulating host immunometabolism—paving transformative avenues against recalcitrant intracellular infections.

Currently, the understanding of how different designs influence anti-infective efficacy remains superficial. Targeted delivery can increase intracellular drug concentrations, temporarily overcoming drug resistance mechanisms such as efflux pumps. However, long-term selective pressure may drive pathogens to evolve new escape strategies, such as downregulating mannose receptor (MR) expression on host cells, altering intracellular survival niches, or enhancing drug metabolism. Most current studies focus on short-term efficacy evaluations in acute infection models, lacking monitoring and consideration of the evolution of drug resistance during long-term treatment. Anti-infective therapy faces distinct challenges compared to cancer treatment: infection sites are often accompanied by intense inflammation, edema, necrosis, and tissue remodeling, which significantly affect the extravasation, diffusion, and cellular uptake of nanocarriers. Current models often simplify this complexity. Macrophages and other cells targeted by mannosylated nanocarriers act not only as “delivery vehicles” for drugs but also as “effectors” of host defense. How nanocarriers and drugs affect the functions of these cells (e.g., phagocytosis, bactericidal activity, cytokine secretion)—whether enhancing or interfering with innate immunity—requires careful evaluation. Inappropriate modulation may lead to immunopathological damage or chronicity of infection. Many of the infectious diseases under study primarily affect resource-limited regions. Despite the technical advantages of mannose targeting, complex nanoformulations may pose challenges in terms of high costs, production, storage, and distribution. While pursuing technological innovation, it is imperative to consider their real-world accessibility.

### 6.3 Application of mannosylated nanocarriers in imaging

Tumor-associated macrophages (TAMs) are key regulators within the tumor microenvironment (TME), playing a central role in promoting tumor growth, immune evasion, and therapy resistance. However, studying their functional heterogeneity and dynamic behavior has been challenging due to the lack of effective methods for *in vivo*, dynamic monitoring of TAMs. To overcome this bottleneck, molecular imaging strategies targeting the mannose receptor (CD206), which is highly expressed on the surface of TAMs, have emerged. Recent cutting-edge research has focused on developing mannose-based nanoprobes to achieve non-invasive and precise visualization of TAMs. This aims to provide deeper insights into their roles in tumor progression and treatment response, while also offering new perspectives and tools for developing targeted therapies.

A novel imaging strategy was developed by Li Y. et al. (2020), utilizing anti-biofouling PEG-b-AGE polymer-coated iron oxide nanoparticles functionalized with mannose on their surface to construct a probe targeting the mannose receptor on M2-type TAMs. *In vitro* experiments demonstrated a high co-localization rate of 94.7% between this probe and tumor tissue sections. In tumor-bearing mouse models, at 48 h post intravenous injection, the targeted probe induced a significantly greater decrease in T2 relaxation time in the tumor region compared to the non-targeted probe. Further immunofluorescence analysis confirmed that Man-IONPs could specifically target CD206+ M2 macrophages in various regions of the tumor at different time points, whereas non-targeted nanoparticles were cleared from the tumor within 18 h. This study indicates that anti-biofouling mannosylated iron oxide nanoparticles enable specific *in vivo* imaging of M2-type TAMs, providing a powerful tool for investigating macrophage dynamics within the tumor microenvironment and for developing targeted therapies.

Gu et al. (2023) developed a radionuclide gallium-68-labeled mannosylated serum albumin ([^68^Ga]Ga-MSA) nanoparticle, aiming to non-invasively monitor the immune status of the tumor microenvironment (TME) by targeting the mannose receptor on the surface of macrophages. Research results indicated that in mouse models responsive to anti-PD-1 therapy, the proportions of both tumor-associated macrophages (TAMs) and lymphocytes within the TME significantly increased, with TAMs being the most abundant immune cell population among them. Positron emission tomography (PET) imaging further confirmed that during the early stages of treatment, tumor sites in responders exhibited significantly higher uptake of [^68^Ga]Ga-MSA, suggesting an increase in TAM numbers. It was concluded that non-invasive imaging of TAMs using MSA nanoparticles can effectively reflect the immune-enriched status of the TME, which is closely associated with the efficacy of anti-PD-1 therapy, thereby offering a highly promising new method for monitoring the effectiveness of immune checkpoint inhibitors.

Based on the high expression of the mannose receptor on TAMs, Locke et al. (2012) designed a mannosylated liposome. This probe offers unique advantages: its hydrophilic core encapsulates the radionuclide ^64^Cu for PET imaging, while its lipid bilayer is embedded with a fluorescent dye to facilitate subsequent microscopic observation, enabling multimodal tracking. In a mouse model of lung cancer, PET imaging at 6 h post intravenous injection showed that the mannosylated liposomes specifically accumulated in TAMs at the tumor site, with minimal accumulation in distant normal lung tissue. The study demonstrates that this mannosylated liposome is a promising carrier capable of efficiently delivering imaging agents to pulmonary TAMs. This platform not only provides a powerful tool for the non-invasive assessment of TAMs and understanding their role in tumor progression but also shows significant potential for delivering therapeutic agents to the tumor microenvironment.

In the field of infectious disease prevention and control, the precise localization of infection foci is a major challenge. Many pathogens (e.g., *mycobacteria*, fungi) can be phagocytosed by macrophages and establish long-term latency within these cells, forming “sanctuaries” (Zhang W. et al., 2018) that are difficult to detect with conventional imaging techniques, often leading to infection recurrence and treatment failure. Given that activated macrophages highly express the mannose receptor (MR, CD206) on their surface, mannosylated nanocarriers have emerged as intelligent targeting probes. This strategy involves efficiently delivering imaging agents (e.g., radionuclides, fluorescent dyes) to macrophages at the infection site, paving a new avenue for non-invasive, high-sensitivity imaging and differential diagnosis of deep-seated and occult infections.

Addressing the challenge of treating intracellular *Methicillin-resistant Staphylococcus aureus* (MRSA) infections, where antibiotics often fail to effectively penetrate cell membranes, Peng H. et al. (2022) constructed a multifunctional antibiotic delivery system (WP5 ⊃ G) based on host-guest interactions between mannose-modified pillar [5]arene (WP5) and a near-infrared fluorescent guest molecule (G). This system can encapsulate hydrophobic antibiotics like linezolid to form nanoparticles (LZD–WP5 ⊃ G). These nanoparticles target macrophages via mannose receptor-mediated endocytosis and trigger antibiotic release in the intracellular high-concentration glutathione (GSH) environment, significantly enhancing their antibacterial activity against intracellular MRSA. This system exhibits good biocompatibility and straightforward preparation. Furthermore, it enables real-time tracing of the drug release process and non-invasive cellular imaging through a near-infrared fluorescence “off-on” response, offering a novel strategy for developing new intelligent delivery platforms targeting intracellular infections. This targeting paradigm extends beyond oncology. For atherosclerosis theranostics, Lee et al. (2024) designed macrophage-targeted photoactivatable nanoagents (MAN-PEG-Ce6). Atherosclerotic plaques harbor pro-inflammatory macrophages—key drivers of plaque instability. MAN-PEG-Ce6 precisely targets these cells, enabling:Fluorescence imaging of lesions via Ce6 emission (diagnosis)ROS-mediated ablation of foam cells and pro-inflammatory macrophages (therapy).This integrated strategy suppressed atherosclerotic inflammation while demonstrating safety in dermal and other tissues, highlighting its translational potential for precision phototherapy of cardiovascular diseases.

In summary, mannosylated nanocarriers have emerged as a highly promising targeted platform, with successful applications spanning three major fields: oncology, infectious diseases, and cardiovascular diseases. In oncology, this technology enables high-specificity imaging to reveal the dynamics of Tumor-Associated Macrophages (TAMs) and their response to immunotherapy. For infectious diseases, it achieves precise localization of hidden infection foci and enables intelligent drug delivery. In the context of atherosclerosis, it demonstrates significant theranostic potential. Collectively, these studies corroborate that targeting the macrophage mannose receptor can overcome the limitations of traditional diagnostic and treatment models. This approach provides a unified technological paradigm for the precise visualization and effective treatment of a series of major diseases, showcasing broad clinical translation prospects.The limitations of different nanocarriers and their improvement strategies are summarized in Table 3.

**TABLE 3:** Limitations and improvement strategies of different nanocarriers.

Nanocarrier type	Limitations/Challenges	Improvement strategies
Lipid-based nanocarriers (e.g., Liposomes, SLNs, NLCs)	1. Poor stability (prone to aggregation, oxidation) 2. Prone to premature drug leakage 3. Limited drug loading capacity 4. Rapid clearance by the immune system (e.g., RES/MPS)	1. Surface PEGylation (to create “stealth” liposomes) 2. Active targeting modification (e.g., conjugation of antibodies, ligands) 3. Stimuli-responsive design (e.g., pH-, temperature-sensitive) 4. Process optimization (e.g., lyophilization to improve stability)
Polymer-based nanocarriers (e.g., Nanoparticles, Micelles, Dendrimers)	1. Potential toxicity of polymer materials 2. Non-uniform drug distribution and low loading capacity 3. *In vivo* instability and premature dissociation of micelles 4. Cationic toxicity of dendrimers (due to positive surface charge) 5. Complex synthesis process	1. Use of biodegradable materials (e.g., PLGA) 2. Cross-linking of micelles (to enhance stability) 3. Surface modification (e.g., PEGylation, acetylation to reduce toxicity) 4. Development of novel synthesis methods (e.g., click chemistry)
Inorganic nanocarriers (e.g., Mesoporous silica, Gold, Magnetic nanoparticles)	1. Poor biodegradability, leading to long-term retention 2. Unknown long-term toxicity potential 3. Prone to aggregation 4. Difficult to clear from the body	1. Surface functionalization/coating (to improve biocompatibility and stability) 2. Precise control over size and morphology 3. Development of biodegradable inorganic materials 4. Design as theranostic agents (to leverage their inherent imaging capabilities)
Biogenic nanocarriers (e.g., Exosomes, Albumin nanoparticles)	1. Difficulties in isolation and purification, resulting in low yield 2. Low drug loading efficiency 3. High batch-to-batch heterogeneity 4. Potential immunogenicity risk	1. Engineering modifications (to introduce targeting capabilities) 2. Development of efficient drug loading techniques (e.g., electroporation, sonication) 3. Optimization of cell-based production systems for scale-up 4. Establishment of standardized protocols for quality control

### 6.4 Synergistic effects

Mannosylated nanocarriers exhibit multidimensional advantages in combination therapy. Beyond serving as a targeting moiety, mannose can also directly interfere with tumor metabolism: Enhanced glucose uptake—a hallmark metabolic alteration in many cancers known as the Warburg effect—reveals a metabolic vulnerability in tumor cells (Hanahan and Weinberg, 2011; Pavlova and Thompson, 2016). Mannose accumulates as mannose-6-phosphate in tumor cells, which effectively blocks the glycolytic pathway by inhibiting hexokinase and phosphoglucose isomerase, thereby suppressing tumor growth (Gonzalez et al., 2018). This unique mechanism endows mannose with a more significant tumor growth inhibitory effect compared to other monosaccharides.

Based on this property, researchers have developed a variety of intelligent nano-delivery systems. Yuan et al. constructed pH-sensitive mannose-polymer nanoparticles containing tertiary amine groups, co-loading the photosensitizer BDPI. In the acidic tumor microenvironment, tertiary amine protonation and Schiff base cleavage synergistically trigger the dual release of mannose and the photosensitizer, achieving synergistic enhancement of metabolic inhibition and photodynamic therapy (Yuan et al., 2019). The mannose-curcumin solid lipid nanoparticles developed by the Chae team bind to mannose receptors on the surface of lung cancer cells and macrophages with high affinity, simultaneously achieving dual antitumor and antibacterial effects (Chae et al., 2024).

In terms of constructing combination therapy systems, Yang et al. (2025) designed a mannose-modified self-assembled nanosystem (SeBDP@TPZ-S-S-Cy/Man NPs), which cleverly integrates targeted delivery and therapeutic synergy mechanisms. This system connects TPZ and Cy3 via GSH-responsive bonds and encapsulates the photosensitizer SeBDP. Mannose modification endows it with A549 tumor-targeting ability; in the intracellular high-GSH environment, rapid drug release triggers a cascade reaction: SeBDP consumes oxygen to generate reactive oxygen species (ROS) through photodynamic action, exacerbating tumor hypoxia, which in turn activates the conversion of TPZ into cytotoxic free radicals, realizing efficient synergy between photodynamic therapy and hypoxia-activated chemotherapy.

These studies indicate that mannose-modified nanocarriers not only improve the targeted delivery efficiency of drugs but also generate synergistic effects with other therapeutic modalities through metabolic intervention, providing a new direction for the development of precise and efficient cancer treatment strategies.

### 6.5 Bio-derived nanoplatform

Bio-derived nano-platforms refer to nano-delivery systems constructed based on natural biological materials such as cell membranes, exosomes, and lipoproteins. Owing to their excellent biocompatibility, low immunogenicity, and inherent targeting ability, they exhibit great potential in disease diagnosis and treatment (Gao et al., 2023; Lin and Wang, 2023). Especially in the field of tumor immunotherapy, such platforms can mimic certain characteristics of pathogens or tumor cells, thereby activating specific immune responses more efficiently. Based on this, researchers are committed to developing novel bio-derived nano-vaccines to address issues of traditional vaccines such as limited immunogenicity and poor targeting.

Melanoma, as a tumor with high immunogenicity, serves as an ideal model for studying cancer immunotherapy. In recent years, vaccine strategies based on nanotechnology have provided new avenues for activating anti-tumor immune responses. An innovative study (Liu et al., 2024) constructed a novel “three-in-one” multi-antigen nanovaccine (TBM) by fusing B16F10 melanoma cell membranes with *E. coliexosomes* using sequential extrusion technology, followed by modification with DSPE-PEG-mannose. The TBM vaccine demonstrated potent immune-activating capabilities. *In vitro*, it not only induced the polarization of RAW264.7 macrophages towards a pro-inflammatory M1 phenotype, triggering the release of cytotoxic factors, but also promoted the maturation and antigen-presenting function of bone marrow-derived dendritic cells (BMDCs), thereby activating splenic T cells and achieving specific killing of B16F10 tumor cells. In animal models, the TBM vaccine significantly inhibited the growth and metastasis of melanoma and extended the survival of mice, showing clear prophylactic efficacy. Furthermore, the research extended this platform to the field of personalized therapy. By integrating cell membranes from autologous melanoma tissues, an autologous TBM (ATBM) vaccine was created. When combined with a low-dose anti-PD-1 antibody, the ATBM vaccine effectively activated anti-tumor immunity and significantly increased the survival rate of mice with allogeneic melanoma transplants.

Methotrexate (MTX) is a representative disease-modifying antirheumatic drug, but conventional treatment regimens have limitations and numerous side effects. To enhance the targeting ability of MTX in rheumatoid arthritis (RA) therapy and prolong its circulation time, a study (Chen et al., 2021) successfully developed a novel drug delivery system—mannose-modified methotrexate human serum albumin nanoparticles (MTX-M-NPs). The researchers first synthesized a mannose-derived carboxylic acid and modified it onto the surface of MTX-NPs. The formulation was systematically optimized using a central composite design method. The final prepared MTX-M-NPs had a particle size of 188.17 ± 1.71 nm and an encapsulation efficiency of 95.55% ± 0.33%. Successful construction of the delivery system was confirmed by nuclear magnetic resonance and Fourier transform infrared spectroscopy. Cellular uptake experiments showed that the uptake rate of MTX-M-NPs in neutrophils was significantly higher than that of unmodified nanoparticles.

#### 6.5.1 Pharmacokinetic

This research lays a solid foundation for the targeted treatment of rheumatoid arthritis, demonstrating the potential of mannose-modified albumin nanoparticles in improving drug delivery efficiency and pharmacokinetic properties.

## 7 Challenges in the in vivo application of nanocarriers

The *in vivo* application of nanoparticles presents several challenges, including: (1) Poor stability in systemic circulation: Nanoparticles may exhibit insufficient stability and consequently aggregate or precipitate in the high-salt, high-protein environment of the blood and its colloidal system (Swinton et al., 2020; Hu et al., 2024). This can lead to targeting failure or even thrombosis. (2) Premature drug release: Unstable or improperly designed carrier materials can cause the drug to leak before reaching the target site. This can also be triggered by influences from enzymes, pH, and serum components in the blood (Cheng et al., 2022; Park et al., 2025). (3) Unpredictable immune responses: Nanoparticles can be recognized as “foreign bodies” by the body’s immune system, such as the mononuclear phagocyte system (MPS). This may activate the complement system, leading to clearance by the immune system (Gong et al., 2020). This may activate the complement system, leading to clearance by the immune system (Dobrovolskaia et al., 2016). (4) Lack of feasibility for large-scale production:Difficulty in scaling up laboratory processes: Precision techniques like microfluidics are often costly and inefficient for industrial-grade production.Significant batch-to-batch variation: It is challenging to ensure complete consistency in key parameters such as particle size, drug loading capacity, and release profile across different batche (Chen et al., 2024).

The targeting design of nanomaterials primarily involves two main strategies: static ligand targeting and dynamic microenvironment-responsive targeting. Static ligand targeting involves immobilizing one or more targeting ligands (such as antibodies, peptides, or small molecules) on the nanoparticle surface. These ligands remain chemically and structurally stable throughout the circulation process, achieving binding through their high affinity for receptors on the surface of target cells (Deng et al., 2024). In contrast, dynamic microenvironment-responsive targeting refers to nanomaterials designed to sense unique physiological signals at the lesion site (e.g., pH, enzymes, reactive oxygen species, temperature) and subsequently undergo physical or chemical changes (such as disassembly, charge reversal, or a shift in hydrophilicity/hydrophobicity). This allows them to release the drug or alter their behavior at the right place and time. Each of these targeting approaches has its own advantages and disadvantages (Liu Y. et al., 2023). Table 4 analyzes and compares the differences between the static ligand targeting and dynamic microenvironment-responsive strategies.

**TABLE 4:** Comparison of static targeting and dynamic microenvironment-responsive strategies.

Comparison aspect	Static ligand targeting strategy	Dynamic microenvironment-responsive strategy
Core mechanism	Active binding driven by molecular recognition	Intelligent response to physicochemical signals
Mechanism of action	Sustained and constant binding	A stimulus-triggered, burst-like change in behavior
Targeting precision	Achieves specific recognition of target cell types with high spatial precision	Accurately differentiates the microenvironmental differences between pathological and normal tissues
Key advantages	1. Based on an intuitive principle with a well-established design 2. Shows high specificity for target cells, such as receptor-overexpressing cancer cells 3. Efficiently mediates cellular uptake/endocytosis	1. Offers broader applicability as it is independent of receptor expression levels 2. Exhibits excellent deep-penetration capability to access the core of the lesion 3. Enables controlled drug release, significantly reducing off-target toxicity and enhancing the therapeutic index
Key limitations	1. Receptor dependency: Therapeutic efficacy is vulnerable to the heterogeneous and varying expression levels of the target receptor 2. The binding-site barrier (BSB) effect: Carriers that bind to perivascular targets can impede the penetration of subsequent carriers into deeper tumor tissues 3. Protein corona interference: The formation of a protein corona can mask the targeting ligands, leading to impaired targeting efficiency or failure 4. Susceptibility to post-endocytic degradation	1. Complex design and challenging synthesis/characterization 2. Insufficient microenvironmental triggers: Some tumors exhibit only a modest pH gradient or inadequate enzyme levels for an effective response 3. Requires precise control over response kinetics: Suboptimal response rates (either too fast or too slow) can compromise therapeutic efficacy

## 8 Advantages and limitations of the mannose-targeted strategy

### 8.1 The advantages of mannose targeted

Compared with traditional targeting ligands such as folic acid, RGD peptides, and antibodies, mannosylated nanocarriers exhibit multidimensional uniqueness in drug delivery systems, with the core lying in the synergistic unity among targeting precision, biocompatibility, and functional adaptability. In terms of targeting mechanism and efficacy, mannose can form high-affinity binding with CD206 receptors highly expressed on the surface of immune cells such as macrophages and dendritic cells, and achieve rapid intracellular delivery through receptor-mediated endocytosis. Unlike folic acid, which is prone to off-target accumulation due to the basal expression of its receptor in normal tissues, the targeting cell spectrum of mannose is more focused on the immune system. As a natural sugar ligand, mannose-modified carrier materials typically possess characteristics of low toxicity and easy degradation. For example, mannose-modified bovine serum albumin nanoparticles exhibit excellent biocompatibility (Li et al., 2009), while antibody-based targeting agents, due to their complex protein structure, are prone to trigger immunogenic reactions. In terms of formulation stability, cholesterol-derived mannose-modified polypeptide-modified lipid nanoparticles (CPSM-LNPs) (Zeng et al., 2025) can maintain stable particle size and encapsulation efficiency after being stored at 4 °C for 30 days, with a translation efficiency significantly higher than that of commercial ALC-LNP. In contrast, peptide-modified carriers often lose their targeting activity due to peptide chain degradation.

More importantly, compared with other carbohydrate/polysaccharide-based targeting ligands, mannose exhibits more comparable differences and complementarities in mechanisms and applications. For instance, in contrast to β-glucan (such as the antibody-β-glucan conjugate AGC reported in the literature), mannose primarily relies on CD206-mediated endocytosis, while β-glucan can activate pattern recognition receptors like dectin-1, exerting a unique “bridging” effect in enhancing the interaction between dendritic cells and tumor cells. The two are functionally complementary in immune activation pathways (Reid et al., 2009; Cao et al., 2023; Wang et al., 2024). Compared with chitosan and its derivatives (e.g., TACTIC and M2pep-CMCS), mannose possesses higher targeting specificity. However, leveraging its cationic properties and ease of functionalization, chitosan-based nanocarriers offer greater material flexibility and functional integration capabilities in achieving tumor microenvironment retention, loading diverse immune agonists (e.g., R848), and reprogramming the phenotype of tumor-associated macrophages (Virmani et al., 2023; Xu et al., 2024). Furthermore, compared with polysaccharide ligands such as hyaluronic acid (targeting CD44) and cyclodextrin derivatives (e.g., R6RGD-CMβCD), mannose demonstrates more distinct targeting selectivity for immune cells, whereas hyaluronic acid and cyclodextrin systems exhibit unique material advantages in direct tumor cell targeting, controlled drug release, and multimodal combination therapy (Chen et al., 2023; Luo et al., 2024).

The core advantage of mannose in targeted delivery systems lies in its high specificity and excellent biocompatibility with immune cells, particularly antigen-presenting cells. Compared with traditional non-carbohydrate ligands, its targeting spectrum is more focused; while compared with other polysaccharide ligands (e.g., β-glucan, chitosan, hyaluronic acid, etc.), mannose is more prominent in specificity but forms functional complementarities with other carbohydrates in multifunctional integration and microenvironment regulation. Therefore, comparing and synergistically designing mannose within the overall framework of carbohydrate ligands is a scientific approach to enhance the efficacy of targeted delivery systems and promote their clinical translation.

### 8.2 Limitations and failures of mannose targeting

Although mannose-targeted strategies show broad prospects in the field of drug delivery, extensive research has also revealed their limitations, contradictory results, and challenges. Objectively understanding these shortcomings is crucial for the optimization and clinical translation of this technology. The main challenges stem from the heterogeneity of target expression, the complex biological environment *in vivo*, and the design factors of the delivery system itself.

Firstly, the expression of the mannose receptor (MR) is not universal or constant, and it varies significantly among different tumor subtypes, disease stages, and patient populations (Martinez-Pomares et al., 2001). This heterogeneity directly leads to uncertainty in targeting efficiency. In the tumor microenvironment, although M2-type tumor-associated macrophages (TAMs) highly express MR, their infiltration degree and phenotype differ greatly across various cancer types and individuals (Kodama et al., 2020). In some tumor models, due to low MR expression levels or receptor saturation phenomena, the uptake efficiency mediated by mannose may be much lower than expected, or even ineffective (Savić et al., 2013). Secondly, endogenous competition and microenvironmental factors significantly affect targeting efficiency. Naturally occurring mannosylated proteins in the body can compete with nanocarriers for binding to MR, thereby weakening the active targeting effect (Houshmand et al., 2020). The unique physiological barriers of the tumor microenvironment (such as high interstitial fluid pressure, dense extracellular matrix) also hinder the sufficient accumulation and penetration of nanocarriers at the lesion site (Zhang B. et al., 2017; Peng L. et al., 2022). Thirdly, the design parameters of the carriers have a non-linear impact on targeting effectiveness. There is a complex structure-activity relationship between ligand density and targeting efficiency; both excessively high and low degrees of mannose modification can lead to reduced targeting efficiency (Chen et al., 2020). Meanwhile, the physicochemical properties of the nanocarriers themselves (such as size, surface charge) also affect their interaction mode with MR and the internalization pathway (Chen et al., 2018). Finally, the multifunctionality of MR may trigger unexpected biological effects. MR is not only involved in the endocytosis process but also plays an important role in immune regulation. The binding of carriers to MR may unexpectedly activate or interfere with natural immune signaling pathways, producing complex biological responses beyond the expected scope of drug delivery (Nguyen et al., 2009; Panagiotou et al., 2020).

### 8.3 Dual ligand targeting and dual cell targeting

The core limitation of the single-mannose strategy is that “it can only target receptors but cannot deal with the complex delivery barriers in the body.” By introducing a second molecule, dual targeting can simultaneously solve the problems of “barrier penetration” and “precise targeting,” significantly improving the delivery efficiency. To address the challenges faced by respiratory mRNA mucosal vaccines, such as the pulmonary mucosal barrier, insufficient targeting of immune cells, and limited induction of mucosal immunity, a dual-modified mRNA lipid nanoparticle (mRNA-LNP-CS + Man) vaccine with chitosan (CS) and mannose (Man) was developed (Yi et al., 2025). *In vitro* experiments showed that this vaccine had good biocompatibility, high transfection efficiency in various cell lines such as dendritic cells, macrophages, and lung epithelial cells. After intratracheal administration, the vaccine remained in the lungs of mice for up to 72 h, could be evenly distributed throughout the lungs and target immune cells within the lungs, inducing strong systemic and mucosal immunity. It demonstrated excellent lung protection against the SARS-CoV-2 D614G pseudovirus and had good safety, providing a new dual-functional modification strategy for the development of respiratory mRNA mucosal vaccines. To address the issues of high gastric toxicity and irregular intestinal absorption of the colon cancer chemotherapy drug 5-fluorouracil (5-Fu) when administered orally, a dual-targeting oral anticancer delivery system (Poursadegh et al., 2024) based on mannose (Man)-functionalized hydroxyapatite (HAP)/metal-organic framework [MIL-88 (Fe)]-hyaluronic acid (HA) hydrogel film was developed. In HT29 colon cancer cells, this system achieved dual targeting by targeting the MBL receptor with Man and the CD44 receptor with HA, showing high cell uptake and strong cytotoxicity (cell survival rate of approximately 40% at a concentration of 500 μg/mL after 48 h), and low toxicity to normal intestinal epithelial cells, providing a safe and efficient new drug delivery platform for oral chemotherapy of colon cancer. The dual-targeting strategy, based on mannose targeting, introduces a second molecule with “barrier penetration, environmental responsiveness, or additional targeting” functions (such as chitosan CS, hyaluronic acid HA, or *Lactobacillus* biofilm LRB), forming a “multi-linkage synergistic” delivery system. It simultaneously addresses the problems of “barrier penetration” and “precise targeting.” However, this emerging strategy still has certain limitations. Firstly, the manufacturing process is more complex and the economic cost is significantly higher. Secondly, in the dual-targeting system, the two molecules may have physical or chemical interactions, affecting efficacy or increasing toxicity, which requires further long-term research. Most importantly, the efficacy of the dual-targeting strategy is highly dependent on the “expression levels of dual receptors on target cells” and the “functional synergy of the two molecules.” If the target expression is abnormal or the synergy is insufficient, the efficacy may be lower than that of the single-mannose strategy, and individual differences are significant. Another emerging strategy is dual-cell targeting, which centers on “co-regulation of tumor cells and tumor microenvironment (TME)” in tumor treatment (Zhao et al., 2017). It simultaneously targets two types of cells: drug-resistant cancer cells (such as HCT8/ADR) and tumor-promoting M2-type macrophages (TAM). Through dual-targeting, it aims to achieve the dual therapeutic goals of “killing tumor cells + reshaping TME.” It relies on the dual targets of secreted protein acidic and rich in cysteine (SPARC) and mannose receptor (MR) for precise treatment, demonstrating core advantages of “precision and efficiency, multi-mechanism synergy, and low toxicity and safety.” However, its clinical translation still faces limitations such as “controversies over target relevance, ambiguous synergy mechanisms, technical bottlenecks in large-scale production, and insufficient assessment of normal cell impact.” Further research (such as optimizing target validation, clarifying synergy mechanisms, and developing large-scale production processes) is needed to advance it from animal experiments to clinical application.

Future research should pay more attention to evaluating the MR expression profile in disease-specific models to achieve precise patient stratification. At the same time, further optimization of carrier design is needed, such as precisely regulating ligand density, developing condition-responsive release systems, and exploring combined targeting strategies to overcome heterogeneity and biological barriers. Combining mannose-targeted strategies with other treatment modalities (such as immune checkpoint inhibitors) may synergistically overcome the immunosuppressive nature of the tumor microenvironment and bring better therapeutic effects. By facing these challenges directly, mannose-targeted technology can realize its true potential in precision medicine.

## 9 Conclusions and perspectives

Mannose-functionalized nanocarriers, as an active targeting strategy, have evolved over the past decade from a relatively linear drug delivery concept into a highly integrated precision therapeutic platform with multifunctional biological capabilities. This review systematically demonstrates that by specifically “hijacking” the mannose receptor (MR) pathway—overexpressed on tumor-associated immune cells, subsets of malignant cells, and pathogen-infected host cells—this strategy not only provides a precise “navigation system” for conventional chemotherapeutics but also seamlessly integrates with cutting-edge technologies (e.g., phototherapy, gene therapy, immunotherapy), revealing transformative potential in oncology and anti-infective applications.

Evolution from Static Targeting to Dynamic Synergy. Unlike many targeting ligands serving merely as molecular “anchors,” mannose frequently assumes dual or multiple roles. Beyond being a targeting “key,” it possesses intrinsic bioactivity: in oncology, free mannose directly disrupts tumor glycolytic metabolism, enabling metabolic-chemotherapeutic synergy with payloads; in anti-infective therapy, it competitively blocks pathogen adhesion to host cells. This endogenous ligand-payload cooperativity represents a unique strategic advantage.

Advancement from Universal Delivery to Intelligent Responsiveness. Early research focused on constructing stable mannosylated carriers for target-site accumulation. Current frontiers prioritize designing “smart” systems responsive to pathological microenvironments (e.g., tumor acidity, hyper-reducibility, specific enzyme overexpression, or infectious foci acidosis). These platforms enable stimuli-triggered drug release, therapeutic activation, or structural reorganization (e.g., cascade-targeting mechanisms), significantly enhancing spatiotemporal precision.

Expansion from Pathogen Eradication to Host Immunomodulation. The profound significance of mannose targeting lies in its focus on immune hubs—primarily APCs like macrophages and dendritic cells. Consequently, these systems transcend mere “payload delivery” to actively “orchestrate the battlefield.” Whether through DNA vaccine/siRNA delivery to “reprogram” immune cells or nanocarrier-mediated modulation of macrophage polarization/metabolism, this strategy pioneers transformative avenues for active immunoregulation in cancer immunotherapy and host-directed therapy (HDT) against chronic infections.

Moving forward, the development of mannose-targeted nanosystems will focus on multifunctional integration. We anticipate the emergence of theranostic platforms that leverage mannose targeting for lesion visualization, subsequently guiding precision phototherapy or stimulus-responsive drug release. Furthermore, synergistic integration with cutting-edge immunotherapies—such as checkpoint inhibitors (e.g., PD-1/PD-L1 antibodies) and CAR-T cell therapies—holds exceptional promise. For instance, reprogramming tumor-associated macrophages (TAMs) within the immunosuppressive tumor microenvironment via mannose-directed drug delivery may reverse resistance to immune checkpoint blockade, thereby reinvigorating endogenous antitumor immunity.

Although mannose-modified nanocarriers demonstrate significant therapeutic potential in preclinical studies, their translation to clinical applications faces a series of critical bottlenecks that require urgent resolution. Previous reviews often provide insufficient discussion on these specific translational challenges. The primary obstacle to clinical translation is the shift from laboratory-scale preparation to Good Manufacturing Practice (GMP)-compliant large-scale production.The synthesis of mannose-modified nanocarriers involves multiple chemical reactions and purification steps, demanding stringent control over processes, sterility assurance, and environmental monitoring. To achieve scalable and standardized production, research must explore continuous flow manufacturing processes and microfluidic technologies. For instance, employing microfluidic technology allows precise control over the physicochemical properties of nano-formulations, enabling high-throughput and automated production, thereby enhancing process consistency and reliability. Furthermore, establishing the principles of linear scale-up and perfecting the process validation system are crucial for ensuring that the critical quality attributes (e.g., particle size, polydispersity index, drug loading capacity) of the nano-formulations remain consistent from laboratory development to industrial-scale manufacturing. Moreover, the complex composition of nanomedicines often leads to high production costs, which may limit their accessibility. Therefore, conducting cost-effectiveness analyses and optimizing processes to improve yield and reproducibility are extremely important. Strategies such as using high-purity starting materials, optimizing buffer and solvent formulations, and developing technologies for raw material recovery and reuse can help reduce raw material waste and production costs. Another critical aspect is drug reproducibility, which is influenced not only by the production process but also by batch-to-batch variations in raw materials (e.g., polymer molecular weight distribution, degree of mannose modification). Establishing stringent quality control standards, including rigorous characterization of parameters like particle size distribution, zeta potential, drug loading, and release kinetics, forms the foundation for ensuring batch-to-batch reproducibility. Preclinical studies typically show that mannose-modified nanocarriers have good biocompatibility. However, their long-term *in vivo* fate, chronic toxicity potential, and immunogenicity still require systematic evaluation. This includes assessments of cytotoxicity, organ toxicity (particularly focusing on major metabolic and accumulation organs like the liver, kidneys, and lungs), and systemic toxicity (such as hematotoxicity and immunotoxicity). Special attention is needed regarding the mannose receptor (MR) itself, which is involved in immune regulation. The binding of the nanocarrier to the MR could potentially inadvertently activate or interfere with innate immune signaling pathways, triggering complex immune responses. Therefore, immunogenicity assessment (including detection of humoral and cellular immune responses) should be an integral part of long-term safety evaluation. To date, publicly available clinical trial data on mannose-modified nano-delivery systems remains very limited, highlighting the significant gap between preclinical research and clinical application. Most positive results originate from highly optimized animal models, while “negative results” – those failing to replicate expected targeting efficiency/efficacy–are often underreported or not disclosed in academic literature. For instance, some studies might observe toxicity signals due to non-specific aggregation of carriers or unexpected biodistribution, or cases where therapeutic efficacy is inferior to non-targeted carriers because of design complexities (e.g., inappropriate mannose modification density). These “failure” experiences are equally valuable for the healthy development of the field but are rarely disclosed in detail.

In the process of promoting the clinical transformation of glycerol sugar functionalized nanocarriers, it is essential to pay attention to the possible social and ethical impacts. The high treatment costs may limit accessibility in low- and middle-income regions and exacerbate medical inequality; the safety and environmental residue risks of new nanomedicines also pose higher requirements for the regulatory system; moreover, issues such as data privacy and informed consent in individualized treatment also need to be improved in the ethical governance framework while technological development is ongoing, to ensure that the innovative achievements benefit the entire society.

In summary, mannose-modified nanocarriers—empowered by their unique biological targeting mechanisms, multifunctional synergies, and increasingly sophisticated designs—have established a robust precision-guided armamentarium against cancer and infectious diseases. Despite persisting challenges, deepening insights into nano-bio interactions and continuous innovations in materials science will amplify the role of this strategy in advancing precision medicine. Ultimately, it promises to deliver paradigm-shifting therapeutic modalities for combating critical human diseases.
